# Endothelial cell CD36 regulates membrane ceramide formation, exosome fatty acid transfer and circulating fatty acid levels

**DOI:** 10.1038/s41467-023-39752-3

**Published:** 2023-07-07

**Authors:** V. S. Peche, T. A. Pietka, M. Jacome-Sosa, D. Samovski, H. Palacios, G. Chatterjee-Basu, A. C. Dudley, W. Beatty, G. A. Meyer, I. J. Goldberg, N. A. Abumrad

**Affiliations:** 1 Department of Medicine, Division of Nutritional Sciences, Washington University School of Medicine, St. Louis, MO 63110 USA; 2 Department of Microbiology, Immunology, and Cancer Biology, University of Virginia, Charlottesville, VA 22908 USA; 3 Department of Microbiology, Washington University School of Medicine, St. Louis, MO 63110 USA; 4 Departments of Physical Therapy, Neurology and Orthopedic Surgery, Washington University School of Medicine, St. Louis, 63110 USA; 5 Department of Medicine, Division of Endocrinology, Diabetes and Metabolism, New York University Grossman School of Medicine, New York, NY 10016 USA; 6 Department of Cell Biology and Physiology, Washington University School of Medicine, St. Louis, MO 63110 USA

**Keywords:** Fatty acids, Lipid signalling, Actin

## Abstract

Endothelial cell (EC) CD36 controls tissue fatty acid (FA) uptake. Here we examine how ECs transfer FAs. FA interaction with apical membrane CD36 induces Src phosphorylation of caveolin-1 tyrosine-14 (Cav-1Y14) and ceramide generation in caveolae. Ensuing fission of caveolae yields vesicles containing FAs, CD36 and ceramide that are secreted basolaterally as small (80–100 nm) exosome-like extracellular vesicles (sEVs). We visualize in transwells EC transfer of FAs in sEVs to underlying myotubes. In mice with EC-expression of the exosome marker emeraldGFP-CD63, muscle fibers accumulate circulating FAs in emGFP-labeled puncta. The FA-sEV pathway is mapped through its suppression by CD36 depletion, blocking actin-remodeling, Src inhibition, Cav-1Y14 mutation, and neutral sphingomyelinase 2 inhibition. Suppression of sEV formation in mice reduces muscle FA uptake, raises circulating FAs, which remain in blood vessels, and lowers glucose, mimicking prominent *Cd36*^*−/*−^ mice phenotypes. The findings show that FA uptake influences membrane ceramide, endocytosis, and EC communication with parenchymal cells.

## Abstract (web summary)

Endothelial cell CD36 controls tissue fatty acid uptake. Here the authors show how fatty acid uptake by endothelial cells involves regulation of membrane ceramide production, caveolae dynamics, and exosome generation, these events facilitate transfer of circulating fatty acids to tissues and communication between endothelium and parenchyma.

## Introduction

Long-chain fatty acids (FAs) play a prominent role in membrane structure and function. They are important in systemic energy metabolism, with mitochondrial FA oxidation serving as an efficient ATP source for heart and muscle, while FA esterification into triglycerides confers energy storage capacity to adipose and other tissues. Fatty acid oxidation also influences health of stem cells and tissue renewal^1–3^. Fluctuations in cellular FA uptake and utilization during the physiological transitions in energy metabolism are critical for maintaining homeostasis as the circulating FAs are routed to specific tissues^4,5^. During fasting or exercise when glucose supply is reduced, adipose tissue lipases mobilize FAs from stored triglycerides (TGs) to increase supply of circulating FAs. Simultaneously, heart and muscle tissues upregulate FA uptake and oxidation while reducing glucose utilization thus sparing glucose reserves. In contrast, as feeding makes more glucose available, its usage by heart and muscle rises and more FAs are directed for storage in adipose depots^5,6^.

In tissues, FA uptake occurs mainly at the level of capillaries and begins with FA transfer across microvascular endothelial cells (MECs). Surface high-affinity FA receptors on MECs are required to recognize FAs and work in concert with intracellular FA-binding proteins and FA acyl-CoA ligases to promote rapid lipid accumulation^7–9^. In the circulation, FAs are bound to albumin or esterified in lipoprotein triglycerides. FA release from lipoproteins is mediated by intravascular lipoprotein lipase (LpL) and its endothelial cell (EC) anchor protein Gpihbp1. The level of unesterified FA released by LpL at the EC surface is likely to be higher than that of FA dissociated from albumin in the circulation^10^ but is unknown^11^.

Several proteins influence cellular FA uptake including members of the FA transport (FATP) family, the FA binding proteins (FABPs), the acyl-CoA synthases, and cluster of differentiation 36 (CD36). These proteins can function at different levels of the uptake process involving plasma membrane transfer or intracellular channeling of the FA, as reviewed^12,13^. The plasma membrane glycoprotein CD36 was identified as a FA transporter through affinity labeling with a reactive membrane-impermeable sulfosuccinimidyl derivative of oleic acid, SSO^14^, and its physiological role in tissue FA uptake was demonstrated in rodents and humans^15,16^. In addition to albumin bound circulating FAs, CD36 transfers FAs released by LpL from very low-density lipoproteins (VLDL)^17^, however, FA uptake from chylomicron hydrolysis is CD36-independent^18,19^. Crystal structure of CD36 and family members which includes SR-B1 and Limp2 uncovered a common internal lipid transport tunnel^20^ and long-chain FAs were identified in the tunnel of crystal CD36^21^. The FAs bind in a hydrophobic surface pocket of CD36^22^ that connects to the tunnel based on modeling the crystal structure^23^.

CD36 is highly expressed in the capillary endothelium, where it is part of the gene expression signature that defines the lipid handling of microvascular cells^9,24,25^. Strong evidence derived from EC-specific deletion of various transcription factors supports the gatekeeping function of the endothelium in tissue FA uptake^7,24,26^, and EC-specific deletion of CD36 supports its critical role in the process^9^. Mice lacking CD36 in endothelial cells (EC-*Cd36*^*−/−*^) have impaired FA uptake into adipose tissue, heart, skeletal muscle^9^, and the gastric mucosa^3^. EC-*Cd36*^*−/−*^ mice also recapitulate the higher plasma FAs and enhanced glucose disposal observed in germline *Cd36*^*−/−*^ mice^9^.

In this study, we examined the mechanism by which ECs transfer exogenous FA to parenchymal cells. We document that FAs elicit CD36-dependent phosphorylation of caveolin-1 (Cav-1) and generation of ceramides in caveolae, which associate with caveolae budding into internal vesicles (IVs) containing FA, CD36, Cav-1, and ceramides. These vesicles are subsequently released at the basolateral membrane as exosome-like small extracellular vesicles (sEVs) that deliver FAs to tissue cells. We define the FA-sEV pathway biochemically using various approaches and document it’s in vivo contribution using a reporter mouse with EC expression of fluorescent CD63. Inhibiting the sEV pathway in mice reduces FA uptake by muscle fibers and increases circulating FA levels.

## Results

### Fatty acids are endocytosed apically by endothelial cells (ECs) and secreted basolaterally in exosome-like small extracellular vesicles (sEVs)

**Fig. 1:**
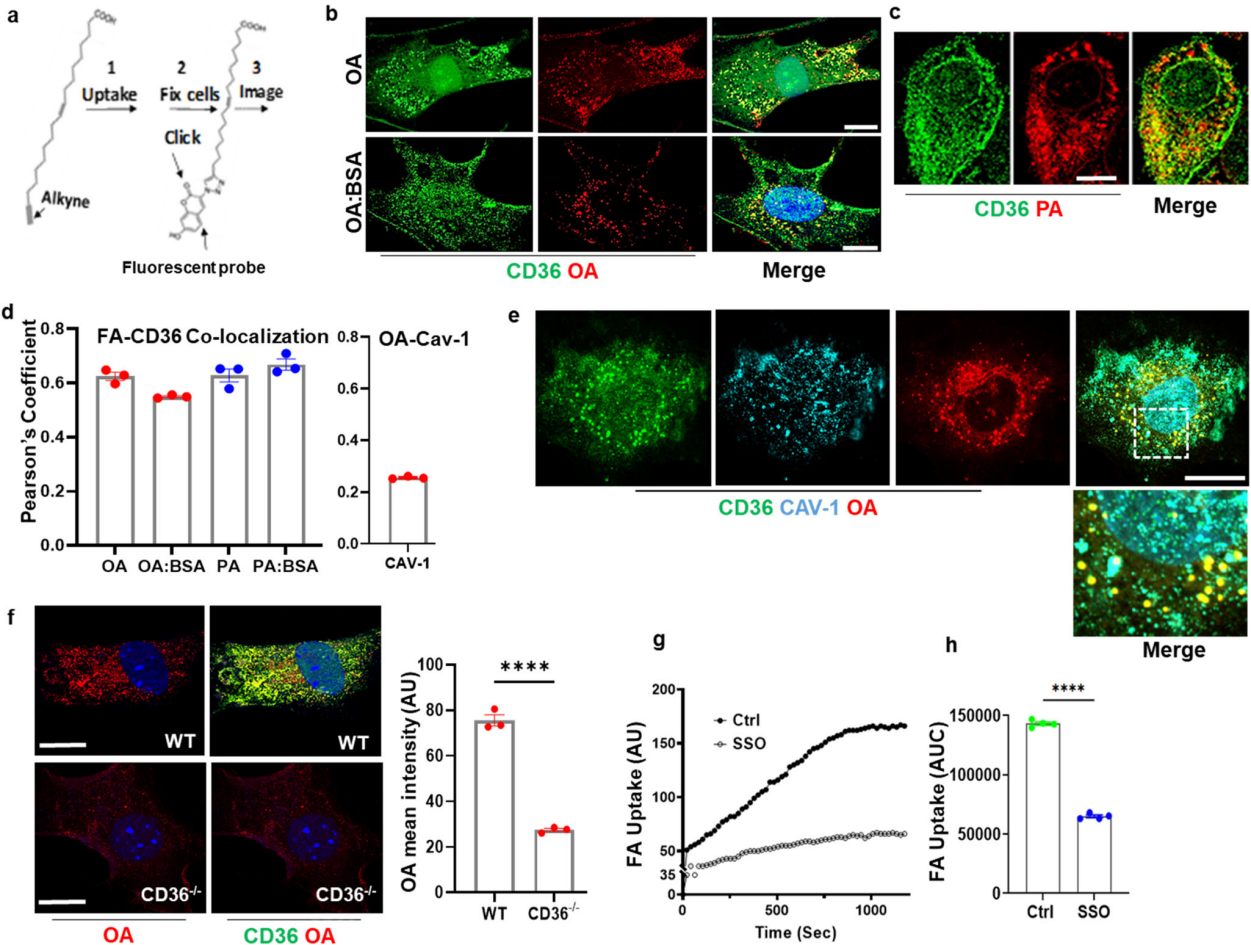
Visualization of oleic acid (OA) and palmitic acid (PA) uptake by microvascular endothelial cells (MECs). **a** Workflow for FA visualization using alkyne FA: Cells were treated with alkyne FAs for the indicated times then fixed and the FAs conjugated to azide coupled with Alexa Fluor via copper-catalyzed click chemistry. **b**, **c** FA internalization in complex with CD36: **b** Primary human-derived microvascular cells (hMECs) were treated with alkyne oleic acid (OA) (15 µM, 10 min) without BSA, upper panel, or with BSA (0.2%), lower panel. **c** Cells treated with PA (15 µM, 0.2% BSA, 10 min). Post uptake, cells were click reacted Alexa Fluor 555 (red), co-stained for CD36, and visualized with anti-goat secondary antibody coupled to Alexa Fluor 488 (green). The visible yellow puncta are positive for OA or PA and CD36. Scale bar: 10 µm. **d** Pearson’s correlation coefficient for FA co-localization with CD36 or Cav-1. Coefficients are high for CD36 and relatively low for Cav-1. *n* = 3 independent experiments, 15–25 cells counted in each set. *p* > 0.05 by two-way ANOVA adjusted for multiple comparisons for FA-CD36 co-localization with/without BSA, left panel. Data are means +/− SEM). **e** Cav-1 is in proximity of intracellular OA/CD36 puncta: mMECs from WT mice were treated 10 min with alkyne OA and co-stained for CD36 and Cav-1. OA (red, Alexa Fluor 555), CD36 (green, Alexa Fluor 488), and Cav-1 (cyan, Alexa Fluor 647). Merge shows Cav-1 (cyan) that associated with CD36-OA yellow puncta, and also unassociated Cav-1. Scale bar: 10 µm. Data are representative of at least three separate experiments. **f**. FA accumulation is reduced in Cd36^−/−^ ECs: Primary mouse lung MECs (mMECs) from WT and *Cd36*^*−/−*^ mice were treated with alkyne OA (15 µM, 10 min). The reduced uptake is quantified in the adjoining graph. *n* = 3 independent experiments, *p* < 0.0001 by unpaired t test, 17–20 cells counted per experiment, Data are means +/− SEM. Scale bar: 10 µm. **g** Real-time FA uptake. Real-time FA uptake in hMECs treated with vehicle (DMSO) or SSO to inhibit CD36, quantified in (**h**) as area under the curve. *n* = 4 independent experiments, ****p < 0.0001 by unpaired t test. Data are means +/− SEM.

We previously reported on critical contribution to tissue FA uptake of EC CD36^9^. CD36 deficient mice show reductions in FA uptake by heart, muscle, fat, and gastric tissues^3,27^ that are replicated by specific deletion of EC CD36^3,9^. Microvascular ECs in heart, muscle, and lung where tissue FA uptake occurs have high expression of CD36^28^, while levels are lower in macrovascular ECs^25^. To investigate the pathway for FA uptake in microvascular ECs (MECs) we used mouse primary ECs (mMECs) isolated from lungs of wild-type or CD36^−/−^ mice^9,29^, and human-derived dermal microvascular ECs (hMECs)^30–32^. These capillary-derived cell types have high expression of CD36^9,24,25^. To trace the FA, we used biologically relevant alkyne-modified analogs of long-chain FAs, amenable to click chemistry that can be reacted post cell fixation with azide-fluorochromes^33,34^ (Fig. 1a). The reactive tag in alkyne-modified FAs is small, consisting of only two carbon atoms connected by a triple bond and results in minimal disruption of cellular FA handling^35–37^. Microvascular ECs were exposed to 15 μM alkyne FAs; oleic or palmitic acids (OA, PA, respectively). Cells were then fixed, the alkyne FAs were conjugated to Alexa Fluor 555 azide (red) followed by immunostaining for CD36 (green). Fatty acid uptake studies traditionally add FAs complexed with bovine serum albumin (BSA) to solubilize the FA and relatively high FA concentrations (>100 µM) are used to keep FA dissociation from becoming limiting^38^. In the present work we used mostly short incubations (10–15 min) and the alkyne FA concentrations found optimal for our microscopic studies were low (10–25 µM), so BSA was not required. We compared uptake of FA complexed to 0.2% BSA to FA without BSA. Omission of BSA did not significantly alter the CD36/FA puncta (Fig. 1b, c). Quantification of CD36-FA co-localization^39^ yielded a strong Pearson’s coefficient of 0.57–0.65 for OA and PA, which was little changed whether BSA was included or not during uptake, as determined by two-way ANOVA adjusted for multiple comparisons (Fig. 1d, *p* > 0.05, *n* = 3 experiments, cells analyzed each experiment = 15–25). Thus, we omitted BSA from our microscopy studies to avoid the factors introduced by BSA FA-binding and BSA transcytosis. However, BSA was kept in the medium during long incubations when higher FA concentrations were needed, as indicated.

Caveolin-1 (Cav-1) was reported to bind FA in adipocytes^40^. Immunostaining for Cav-1 (cyan) in MECs was robust, consistent with its known abundance in microvascular ECs^41^. Cav-1 appeared to dot the CD36-OA puncta (Fig. 1e) but in contrast to the tight co-localization of CD36 and OA, co-localization of Cav-1 and OA was low and had a Pearson’s coefficient of 0.26 (Fig. 1d, *n* = 3 experiments, cells analyzed per experiment = 15). In addition, only some of immunostained Cav-1 (cyan) associated with the OA/CD36 complex (Fig. 1e, insets) consistent with the more ubiquitous role of Cav-1 in EC biology^42^.

Uptake of alkyne OA, quantified by its mean fluorescence intensity, was markedly reduced by CD36 deletion in mMECs of *Cd36*^*−/−*^ mice as compared to mMECs from wild-type (WT) mice (Fig. 1f). Inhibition of CD36 by SSO, which binds the protein irreversibly^22,43^ substantially reduced hMEC FA uptake, measured in real-time using Bodipy-C12 FA, a FA analog that competes well with native FAs^44,45^ (Fig. 1g). The uptake slope was reduced by 70% (0.116 to 0.032) and the area under the curve (AUC) by 65% (Fig. 1h, *p* < 0.0001, *n* = 4 experiments). Together the data in Fig. 1 show that CD36 in MECs recognizes alkyne FA and internalizes the FA with CD36 and Cav-1. Results with both alkyne OA and Bodipy-C12 FA showed CD36 is required for optimal FA uptake by MECs in line with previous findings in mice where deletion of CD36 in ECs reduced tissue FA uptake^9^.

### Role of caveolae in FA uptake by MECs

**Fig. 2:**
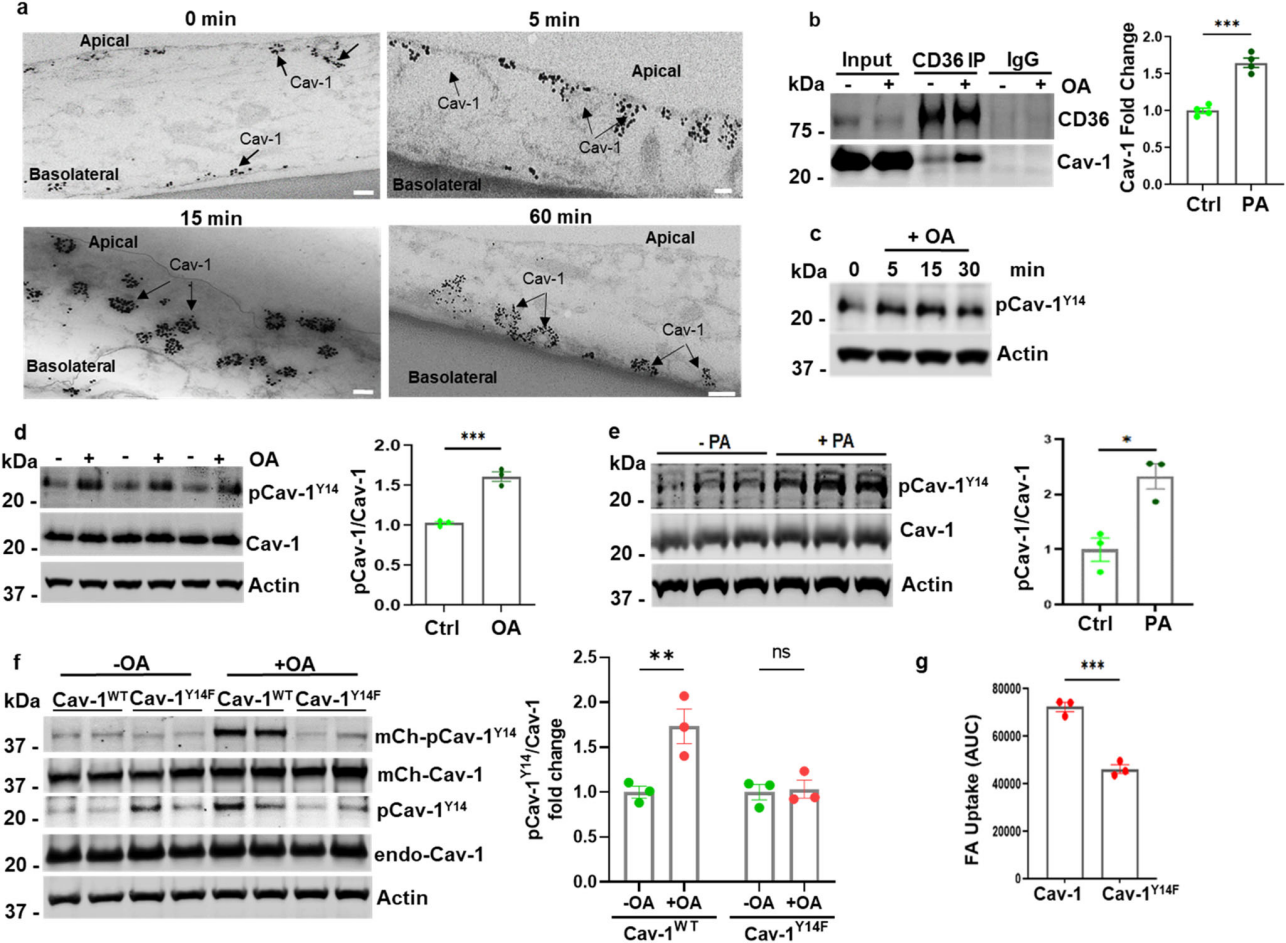
Oleate enhances Caveolin-1 transcytosis and phosphorylation at tyrosine 14. **a** hMECs were serum starved for 4 h and treated with OA (OA-albumin: 100 µM–50 µM) at indicated times, followed by fixation and processing for Cav-1 immune electron microscopy. Scale bars: 100 nm. **b** Serum-starved hMECs (4 h) were treated with OA for 30 min, CD36 was immunoprecipitated (IP) from equal protein lysate and IPs probed for CD36 and Cav-1. Quantification of the increase in CD36/Cav-1 interaction after OA. *n* = 4 independent experiments, ***p < 0.001 by unpaired t test. Data are means +/− SEM. **c**–**e** hMEC were treated as in b with OA (**c**, **d**) or PA (**e**) for 30 min except in (**c**) for the indicated times. Lysates from (**d**) and (**e**) were probed for Cav-1, pCav-1^Y14^, and actin. Graphs quantify ratio of pCav-1^Y14^/total Cav-1 in the presence or absence of FA. *n* = 3 independent experiments for OA or PA, ***p < 0.001 for OA, *p < 0.05 for PA by unpaired t test. Data are means +/− SEM. **f** mCherry-Cav-1^Y14F^ dominant-negative mutant on OA-dependent endogenous Cav-1 phosphorylation. mCherry-Cav-1^WT^ or mutant mCherry-Cav-1^Y14F^ were expressed in hMECs and stimulated with OA. Western blot showing the expressed proteins and their OA-induced phosphorylation at the Y14 residue. Actin was the loading control. Graph quantifies phosphorylation of endogenous Cav-1 (pCav-1^Y14^/Cav-1) in cells expressing WT or mutated Cav-1. *n* = 3 independent experiments, Cav-1^WT^, **p < 0.01; Cav-1^Y14F^, ns by unpaired t test. Data are means +/− SEM. **g** Real-time FA uptake. AUC of FA uptake for cells expressing mCherry-Cav-1^WT^ or mCherry-Cav-1^Y14F^. *n* = 3 independent experiments, ***p < 0.001 by unpaired t test. Data are means +/− SEM.

Cav-1 is integral to the formation and maintenance of caveolae through its interaction with various proteins and lipids^42,46^. The caveolae are dynamic 50–100 nm bulb-shaped plasma membrane (PM) invaginations^47^ abundant in MECs. Caveolae function in transcytosis of macromolecules from the luminal/apical side to the basolateral sub-endothelial space^48,49^. In adipocytes, caveolae were linked to FA uptake^50,51^ and FAs were reported to induce caveolae endocytosis in these cells^50^. Using immune electron microscopy (EM) we monitored Cav-1 trafficking as a marker of caveolae movement in hMECs treated with OA for 5, 15, or 60 min. Addition of OA visibly enhanced Cav-1 internalization at the apical membrane, and Cav-1 movement to the basolateral side was consistent with OA inducing Cav-1 transcytosis (Fig. 2a).

Phosphorylation of Cav-1 at tyrosine14 (Y^14^) by Src kinase is required for caveolae endocytosis^52,53^. In MECs, Src associates with CD36^54^ which functions in regulation of Src signaling^55,56^. We examined whether CD36 interacts with Cav-1. CD36 immunoprecipitates (IP) from hMEC lysates contained Cav-1, and Cav-1 amounts increased in the IP complexes of cells exposed to OA (Fig. 2b, quantified in graph, *p* < 0.001, *n* = 4 experiments). We then tested effect of OA-CD36 interaction on Cav-1^Y14^ phosphorylation. Addition of OA induced a rapid increase in pCav-1^Y14^ in hMECs as compared to untreated cells (Fig. 2c, d, quantified in graph, *p* < 0.001, *n* = 3 experiments). Addition of PA similarly induced Cav-1^Y14^ phosphorylation (Fig. 2e, quantified in graph, *p* < 0.05, *n* = 3 experiments). To examine if Cav-1^Y14^ phosphorylation is required for FA uptake, we overexpressed in hMECs a dominant-negative mCherry-Cav-1^Y14F^ mutant^53,57^ or a control mCherry-Cav-1. Both proteins expressed well (Fig. 2f). Wild-type mCherryCav-1 and endogenous Cav-1 were both phosphorylated by addition of OA. The Cav-1^Y14F^ mutation resulted as expected in loss of OA-induced Y14 phosphorylation of mutated Cav-1, and of endogenous Cav-1, acting as a dominant-negative mutant as previously reported^53^. The Y14F mutant suppressed OA induced endogenous Cav-1 phosphorylation (pCav-1/Cav-1) down to levels in untreated cells (graph, *p* < 0.01, *n* = 3 experiments). Expression of the dominant-negative mCherry-Cav-1^Y14F^ mutant significantly decreased FA uptake as compared to expression of mCherry-Cav-1 (Fig. 2g, *p* < 0.001, *n* = 3 experiments). Together these data show that FA induced phosphorylation of Cav-1^Y14^ is important for MEC FA uptake.

**Fig. 3:**
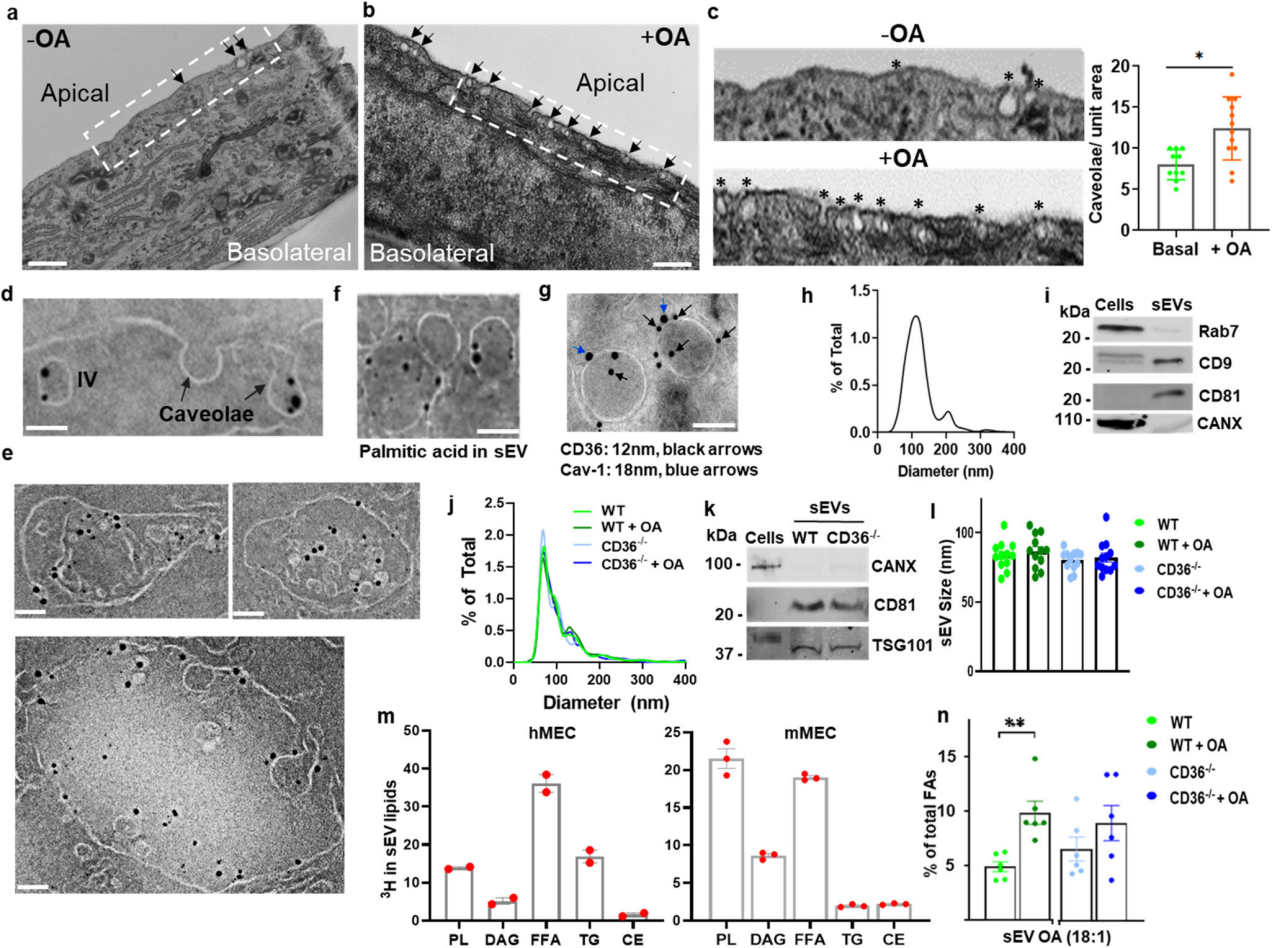
Fatty acid addition enhances caveolae dynamics and induces secretion of small FA-containing extracellular vesicles (sEVs). **a**, **b** Higher number of caveolae at the apical membrane of OA-treated ECs. Serum-starved (4 h) hMEC were treated (10 min) with OA-BSA (100 µM–50 µM), fixed, and examined by transmission electron microscopy (TEM). Scale bar: 500 nm. **c** Magnification of A and B and quantification of caveolae per unit area in OA-treated and control cells. *p < 0.05 by unpaired t test, 10–13 cells analyzed per group. Data are means +/− SEM. **d**, **e** Nanogold palmitic acid (PA) treatment identifies FA in caveolae and multivesicular bodies, MVBs. **d** hMECs treated 10 min with 10 µM nanogold PA (1.14 nm), fixed and processed for EM, show nanogold PA in internalized caveolae and in caveolae undergoing internalization. Scale bar: 100 nm. **e** Nanogold PA identified in the lumen of late endosomes/MVBs. Scale bar: 100 nm. **f** Exosome-like sEVs from ECs incubated with nanogold PA. Scale bar: 100 nm. **g** Immuno EM of sEVs for CD36 and Cav-1: sEVs isolated from hMECs treated with OA:BSA, 100 µM:50 µM, were processed for immune-EM; CD36 12 nm and Cav-1 18 nm. **h**, **i** Size distribution and biochemical markers of exosome-like sEVs secreted by FA-treated MECs: Cells (hMECs) were treated 1 h with OA:BSA, 100 µM:50 µM, and media collected over 24 h and 48 h pooled. The isolated sEVs were analyzed for size distribution (**h**) and probed for exosome markers (**i**) membrane CD9 and CD81, and for exclusion of Rab7, a marker of late endosomes/lysosomes, and CANX an ER marker. Data representative of 3 experiments. **j**–**l** Characteristics of sEVs secreted by WT and CD36^*−/−*^ mMECs in absence or presence of OA. **j** Size distribution, **k** sEV markers. **l** sEV average particle size. *n* = 12 preparations. Data are means +/− SEM. **m** OA distribution (% of total) in sEV lipids from mMECs and hMECS. Cells were incubated 24 h with 50 µM oleic acid containing [^3^H]-oleic acid (22,000 cpm/nmol OA) in culture media supplemented with EV-free FBS. PL, phospholipids; DAG, diacylglycerol, FFA, free fatty acids; TG, triacylglycerol, CE, cholesterol esters. *n* = 2 preparations for hMEC, 3 preparations for mMEC. Data are means +/− SEM. **n** OA is increased in sEVs isolated from wild-type MECs but not in Cd36^*−/−*^ MECs. FA content of sEV by LC/MS lipidomics analysis. *n* = 6 preparations, **p < 0.01 for WT vs WT + OA by one-way ANOVA adjusted for multiple comparisons. Data are means +/− SEM.

The data showing FA induced transcytosis of Cav-1 and the inhibitory effect on FA uptake of the Cav-1^Y14F^ mutant in MECs treated with FA led us to further examine how FA regulate caveolae dynamics using EM. Addition of OA increased the number of caveolae at the apical EC surface or just endocytosed underneath the surface (Fig. 3a–c, quantified in graph as caveolae/unit area, *p* < 0.05, 10–13 cells analyzed per group). When we exposed the cells to nanogold PA we could identify PA in caveolae that are being internalized or that already dissociated forming intracellular vesicles (IVs) (Fig. 3d). The PA was also detected in multivesicular late endosomes also referred to as multivesicular bodies (MVBs) (Fig. 3e, and Supplementary Fig. 1a, b). The MVBs are distinguished by their content of intraluminal vesicles formed by inward budding of the endosome membrane. Vesicles in MVBs can either be degraded by MVB fusion with lysosomes or are released from the cell as small extracellular vesicles (sEV) by MVB fusion with the plasma membrane. The latter outcome was observed as vesicles containing the nanogold PA were released at the EC basolateral side, and these vesicles measured less than 100 nm in diameter consistent with their caveolae origin (Supplementary Fig. 1a, b). To confirm that the internalized FAs are released in sEVs, we isolated sEVs from media of ECs incubated with nanogold PA. Electron microscopy detected PA in sEVs (Fig. 3f) which were also positive for CD36 and Cav-1 using nanogold immunostaining (Fig. 3g).

### Characterization of sEVs

The sEVs secreted by hMECs were isolated and subjected to analysis following the guidelines of the International Society for Extracellular Vesicles, ISEV^58^. The sEVs had an average diameter of about 100 nm (Fig. 3h) and expressed the common sEV (exosome) markers CD9, CD81 and excluded ER calnexin (CANX) and lysosomal Rab7, a marker of late endosome/lysosome and less frequent exosomal marker (Fig. 3i). Size distribution of sEVs from mMECs was broadly similar and did not differ whether cells were or were not treated with OA and if they expressed or did not express CD36 (Fig. 3j). The sEVs isolated from WT or CD36^−/−^ MECs all expressed exosome markers, CD81, and TSG101 related to MVB biogenesis, and excluded CANX (Fig. 3k). No differences were observed in average sEV size (80–90 nm) between WT and CD36^−/−^ mMECs and whether cells were or not OA-treated (Fig. 3L, *n* = 12 preparations).

Distribution of OA in sEV lipids was examined using ^3^H-oleate. The sEVs of human and mouse MECs had, respectively, 34% and 20% unesterified ^3^H-oleate (Fig. 3m, FFA, *n* = 2 experiments). In hMECs sEVs, ^3^H-oleate distributed equally in TGs, (15%) and phospholipids (14%), while in mMEC sEVs, it favored phospholipids (22%) over TG (3%) (Fig. 3m, *n* = 2 experiments). The OA content of sEVs was determined by liquid chromatography–mass spectrometry (LC/MS) and a significant increase in OA from 5 to about 10 percent of total FAs was observed in sEVs from OA-treated WT mMECs as compared to non-treated cells (Fig. 3n, *p* < 0.01, *n* = 6 experiments). This increase was blunted in sEVs from OA-treated CD36^−/−^ mMECs (Fig. 3n, *p* > 0.05, *n* = 6 experiments). Other changes included modest but significant increase in linoleic acid and decrease in docosahexaenoic acid (FA, 22:6) and Caprylic acid (FA, 8:0) in sEVs from WT OA-treated mMECs, but not in sEVs from OA-treated CD36^−/−^ mMECs (Supplementary Fig. 2a, b).

**Fig. 4:**
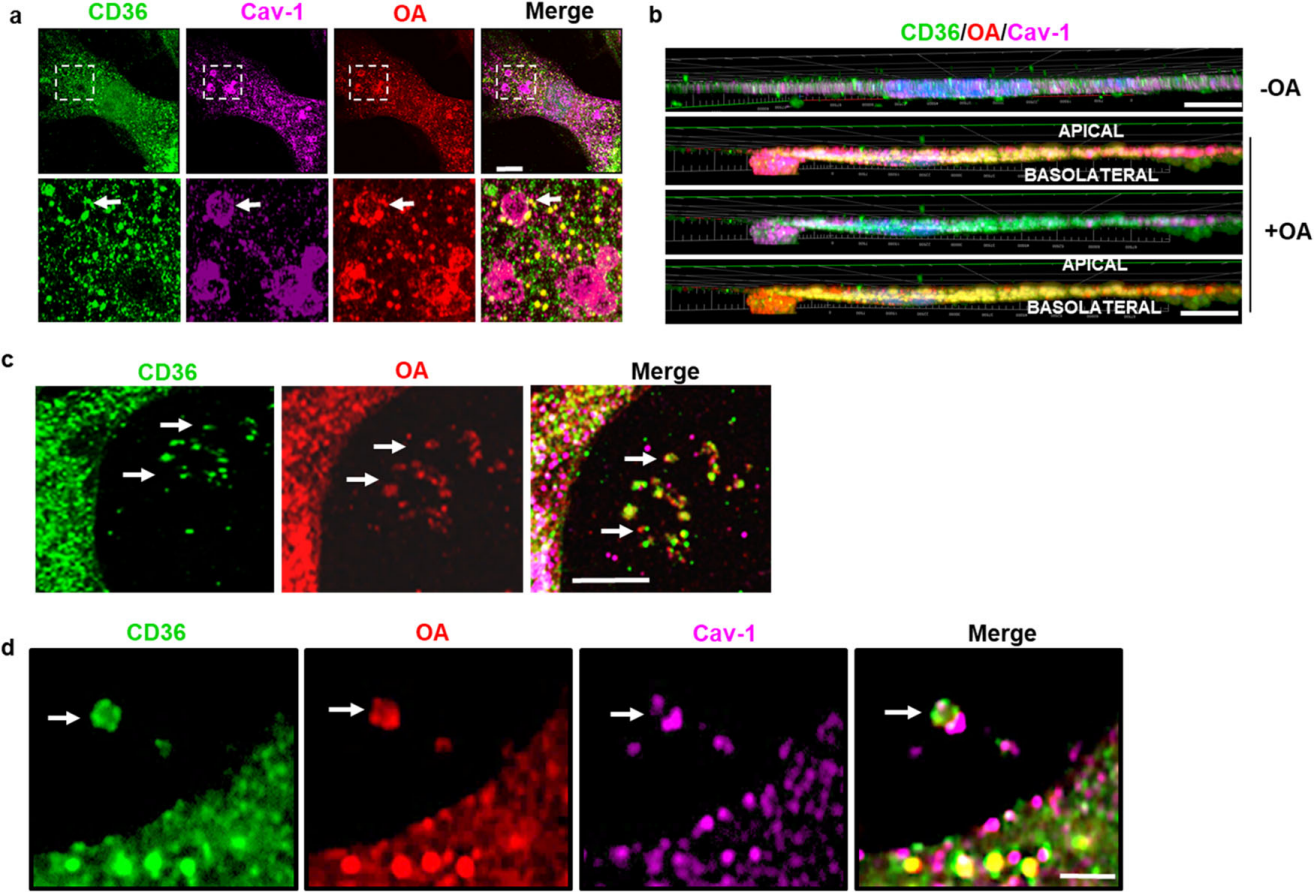
Imaging of endothelial cell basolateral membrane after OA treatment. **a** MVB-like structures at the basolateral EC side, positive for OA, CD36, and Cav-1. hMEC grown to confluence on filters were treated with alkyne OA (15 µM, 10 min). Confocal microscopy identified rosette-like vesicular clusters positive for OA (Alexa 555, red), CD36 (Alexa 488, green), and Cav-1 (Alexa 647, magenta). Scale bar: 10 µm. **b** MVB budding from the EC basolateral side. Three-dimensional rendering of confocal stacks of hMEC images with XZ projections show MVB budding from the membrane at the basolateral side of an OA-treated EC. Scale bar: 10 µm. **c**, **d** Confocal microscopy of sEVs, positive for CD36 (green), OA (red), and Cav-1 (magenta). Scale bars: 1 µm for (**c**), 500 nm for (**d**). Data representative of at least 3 independent experiments.

Visual evidence for presence of CD36, Cav-1, and OA in sEVs was obtained by confocal microscopy of hMECs grown on transwell filters, which allows imaging and visualization of events at the basolateral side of the polarized ECs. In hMECs treated with alkyne OA, three-dimensional rendering of confocal stack images showed rosette-like clusters of vesicles with OA, CD36, and Cav-1 (Fig. 4a) that bud from the basolateral EC side (Fig. 4b), consistent with MVBs fusing with the membrane to release sEVs. In addition, sEVs that are positive for CD36 (green), oleic acid (red), and Cav-1 (magenta) could be observed at the cell periphery and in the adjoining extracellular space (Fig. 4c, d, white arrows). We also immunostained for CD63, a common sEV marker and identified sEVs positive for CD36, CD63, and OA that were released by hMECs in the extracellular space (Supplementary Fig. 1c, d, white arrows).

### CD36 regulation of membrane ceramide mediates FA-sEV secretion

Vesicles generated by endocytosis are either routed to intracellular organelles such as lysosomes or targeted for release into the extracellular space. Ceramides generated from sphingomyelins by neutral sphingomyelinases are important for sorting vesicles into the lumen of MVB for secretion^59^. We immunostained for ceramides^60^ to determine if ceramide generation is involved in OA transcytosis. Antibody specificity was validated by SMase treatment, which enhanced ceramide staining, while staining was eliminated by co-treatment with ceramidase which hydrolyzes ceramides to sphingosine (Supplementary Fig. 3a, b).

**Fig. 5:**
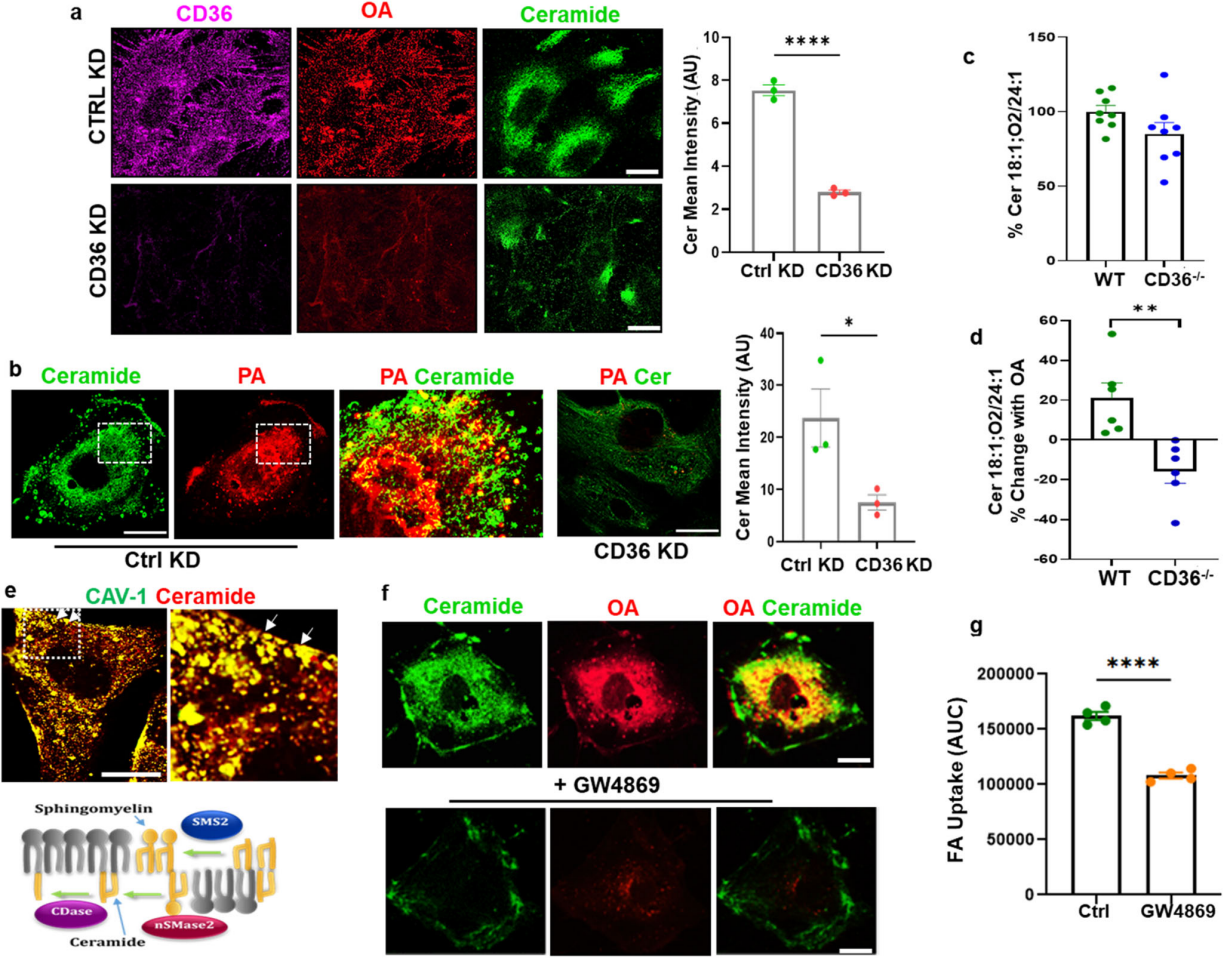
Fatty acids induce ceramide generation in caveolae. **a**–**d** Ceramide generation mediates FA-induced caveolae endocytosis. **a** hMECs, treated with either control siRNA or CD36-specific siRNA, were serum-starved (4 h) and supplemented with alkyne OA (15 µM, 10 min). After the click reaction, cells were immunostained for CD36 and ceramide using secondary antibodies conjugated to Alexa Fluor 647 and 488, respectively. Lower panel, CD36 knockdown (KD) suppresses OA uptake and ceramide formation in hMECs. Adjoining graph, Ceramide mean intensity in control and CD36KD cells. *n* = 3 independent preparations, number of cells counted per preparation: 15–25, ****p < 0.0001 by unpaired t test. Data are means +/− SEM. Scale bars: 10 µm. **b** Alkyne PA treated control or CD36KD hMECs, were immunostained for ceramide. Co-localization of alkyne PA with ceramides is shown. Adjacent graph shows ceramide mean intensity in control and CD36KD cells. *n* = 3 independent experiments, *p < 0.05 by unpaired t test number of cells counted/experiment 10–25. Data are means +/− SEM. Scale bars: 10 µm. **c**, **d** High-performance LC/MS lipidomics analysis on sEVs isolated from mMEC treated with 200 µM OA. **c** Ceramide *Cer 18:1;O2/24:1* content in sEVs from WT and CD36^−/−^ cells was similar. **d** Ceramide *Cer 18:1;O2/24:1* content increased with oleic acid treatment in sEVs from CD36^+/+^ cells and decreased in sEV from CD36^−/−^cells, *n* = 6 independent preparations, **p < 0.01 by unpaired t test. Data are mean values +/− SEM. **e** Ceramide generation in caveolae. Co-localization of ceramide and Cav-1 in OA-treated hMECs: Cav-1 and ceramide are shown to colocalize in plasma membrane caveolae (arrows) and in intracellular vesicles. Scale bar 10 µm. Lower panel in (**e**) shows a cartoon summary of plasma membrane sphingomyelin-ceramide turnover: Neutral sphingomyelinase 2 (nSMase2) localizes to the inner leaflet of caveolae and upon activation generates ceramide, which can be hydrolyzed by ceramidase to sphingosine. Scale bar: 10 µm. **f** The nSMase2 inhibitor GW4869 reduces ceramide formation and OA uptake: hMECs were pre-incubated 10 min with vehicle (DMSO) or GW4869 (10 µM) before adding alkyne OA (15 µM, 10 min). Scale bars: 10 µm. **g** Ceramide formation is critical for FA uptake. Real-time FA uptake (AUC) in hMECs treated with either vehicle or GW4869. *n* = 4 independent experiments, ****p < 0.0001 by unpaired t test. Data are means +/− SEM.

Basal levels of ceramides were barely detectable in MECs in the absence of OA treatment (Supplementary Fig. 3c). Addition of OA rapidly induced ceramide formation in both hMECs (Fig. 5a) and mMECs (Supplementary Fig. 5). Ceramide generation after FA stimulation required CD36 as its knockdown (KD) suppressed OA-induced ceramide formation by 74% (Fig. 5a, quantified in adjoining graph, *p* < 0.0001, *n* = 3 experiments, number of cells counted/experiment 15–25). Ceramide formation in response to OA was similarly blunted in mMECs from *Cd36*^*−/−*^ mice (Supplementary Fig. 5). Like OA, PA induced CD36-dependent ceramide generation (Fig. 5b, quantified in adjoining graph, *p* < 0.05, *n* = 3 experiments, number of cells counted/experiment 10–25). CD36 KD or deletion reduced OA or PA internalization in parallel with suppression of ceramide formation.

The sorting of specific shape-inducing lipids between caveolae and the bulk membrane regulates caveolae formation, stability, and dynamics^61^. We performed targeted lipidomics on the sEVs to examine if OA addition, which induces caveolae endocytosis, associates with noticeable changes in the shape-inducing ceramides and lysophosphatidylcholine (LPC) known to impact caveolae stability^61^. Among the three ceramide species identified in sEVs, we measured Cer 18:1;O2/24:1 previously reported in the few studies that measured sEV ceramides^62^. Basal abundance of Cer 18:1;O2/24:1 was similar in sEVs from WT and CD36^−/−^ mMECs (Fig. 5c, *p* > 0.05, *n* = 8 preparations). Treatment with OA increased levels of Cer 18:1;O2/24:1 in sEVs from WT mMECs while levels were reduced in sEVs from OA-treated CD36^−/−^ mMECs (Fig. 5d, *p* < 0.01, *n* = 6 preparations). We also measured a significant drop in LPC content in sEVs from WT MECs treated with OA and this contrasted with an increase in sEVs from CD36^−/−^ cells treated with OA (Supplementary Fig. 2c, *p* < 0.001, *n* = 10 experiments). These data showed that OA treatment induces opposite changes in ceramide Cer 18:1;O2/24:1 and LPC when sEVs from CD36^+/+^ and CD36^−/−^ mMECs are compared.

Ceramide formation induced by OA addition was observed at cell membrane caveolae invaginations as well as in intracellular vesicles (Fig. 5e, upper panel). Plasma membrane neutral sphingomyelinase 2 (nSMase2) located in the internal leaflet of caveolae^63^ hydrolyzes sphingomyelins to generate ceramide (Fig. 5e cartoon, lower panel) and has been implicated in exosome formation. GW4869 a specific inhibitor of nSMase2 has been widely used to demonstrate nSMase2 involvement in various functions^64–66^. GW4869 prevents enzyme activation by plasma membrane anionic phospholipids such as phosphatidylserine^67,68^ and inhibits exosome secretion^59,69–72^. We found that GW4869 (Fig. 5f) effectively inhibited FA-induced ceramide formation and blocked FA internalization linking ceramide generation by nSMase2 to caveolae endocytosis and FA uptake (Fig. 5f). To further validate these findings, we tested effect of GW4869 on real-time FA uptake. Uptake was significantly reduced in GW4869-treated cells as compared to vehicle-treated controls (Fig. 5g, *p* < 0.0001, *n* = 4 experiments).

**Fig. 6:**
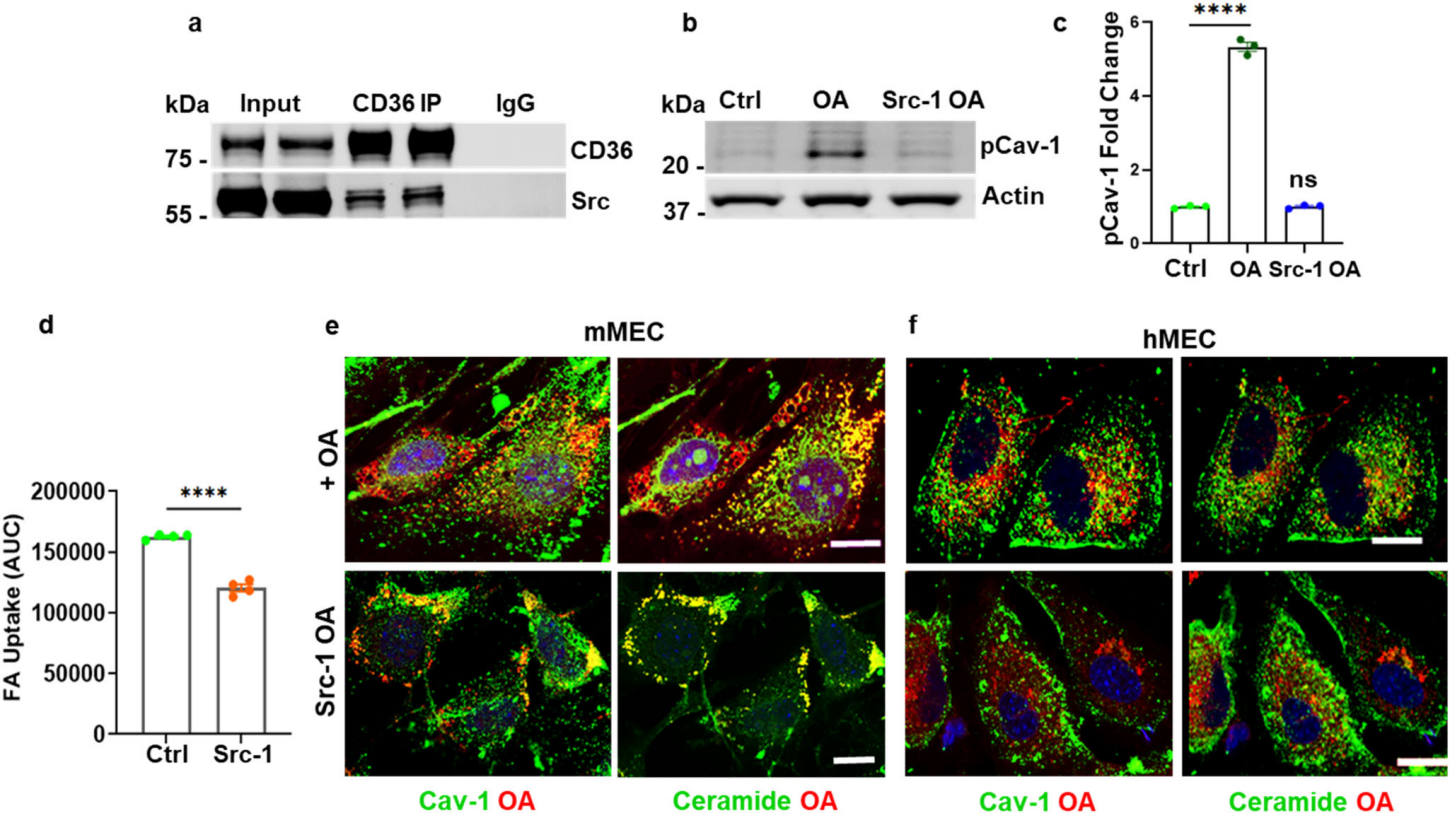
Src Kinase is critical for FA internalization. **a** CD36 interacts with Src in hMECs. CD36 IP shows association of CD36 and Src. **b** Src is critical for OA-induced Cav-1^Y14^ phosphorylation. hMECs were pretreated with Src inhibitor-1 prior to OA addition and lysates were probed for p-Cav-1^*Y14*^. **c** Quantification of (**b**), *n* = 3 independent experiments, ****p < 0.0001 by one-way ANOVA adjusted for multiple comparison, Data are means +/− SEM. **d** Real-time FA uptake (AUC) by hMEC controls or pretreated with Src inhibitor-1. *n* = 4 independent experiments, ****p < 0.0001 by unpaired t test. Data are means +/− SEM. **e**, **f** Src inhibition sequesters oleic acid and ceramide at the cell membrane of mMECs (**e**) or *hMECs* (**f**). Alkyne OA (15 µM, 10 min) was added to ECs without or with pretreatment with Src inhibitor-1 (10 µM, 2 h) before processing for Cav-1 and ceramide immunostaining. Data representative of 3 experiments. Note: Pseudo colors were used to visualize co-localization between OA, Cav-1, or ceramide. Scale bars: 10 µm.

The above data demonstrate that both depletion of CD36 and inhibition of nSMase2 block ceramide generation and FA internalization. We showed earlier in Fig. 2, that OA induces Cav-1 phosphorylation at Y14, a Src kinase site, and this phosphorylation is known to disrupt the caveolae coat promoting caveolae scission from the membrane^53^. We examined the role of Src as CD36 was reported to associate with Src-related protein kinases in MECs^54^. We confirmed the CD36-Src interaction through immunoprecipitation (Fig. 6a). Src inhibition by Src inhibitor-1 blocked OA ability to induce phosphorylation of Cav-1^Y14^ (Fig. 6b, c, *p* < 0.0001, *n* = 3 experiments) and it reduced FA uptake by hMECs measured in real time (Fig. 6d, *p* < 0.0001, *n* = 4 experiments). Furthermore, confocal images of mMECs and hMECs (Fig. 6e, f) pretreated with Src inhibitor-1 prior to adding OA, suppressed FA internalization. The OA and the generated ceramide remained at the cell periphery with Cav-1. Together the data shown in Figs. 1–6 support a molecular mechanism of FA uptake that involves FA interaction with CD36 inducing Src phosphorylation of Cav-1^Y14^ which disrupts the Cav-1 coat and consequently Cav-1 interaction with other caveolae proteins leading to caveolae endocytosis^42,73^. Src did not eliminate ceramide formation in the membrane suggesting that its effect on endocytosis is at least in part independent of nSMase2 activation.

The EC caveolae transcytosis pathway for FAs, was supported by RNASeq data collected in hMECs without and with CD36 KD which showed altered expression of genes related to regulation of caveolae endocytosis (EHD3, Dynamin), vesicular traffic and sorting (VPS37B, RAB36) and vesicle exocytosis (RAB3C) (Supplementary Tables 1 and 2).

### Fatty acid uptake requires FA remodeling of cellular actin

**Fig. 7:**
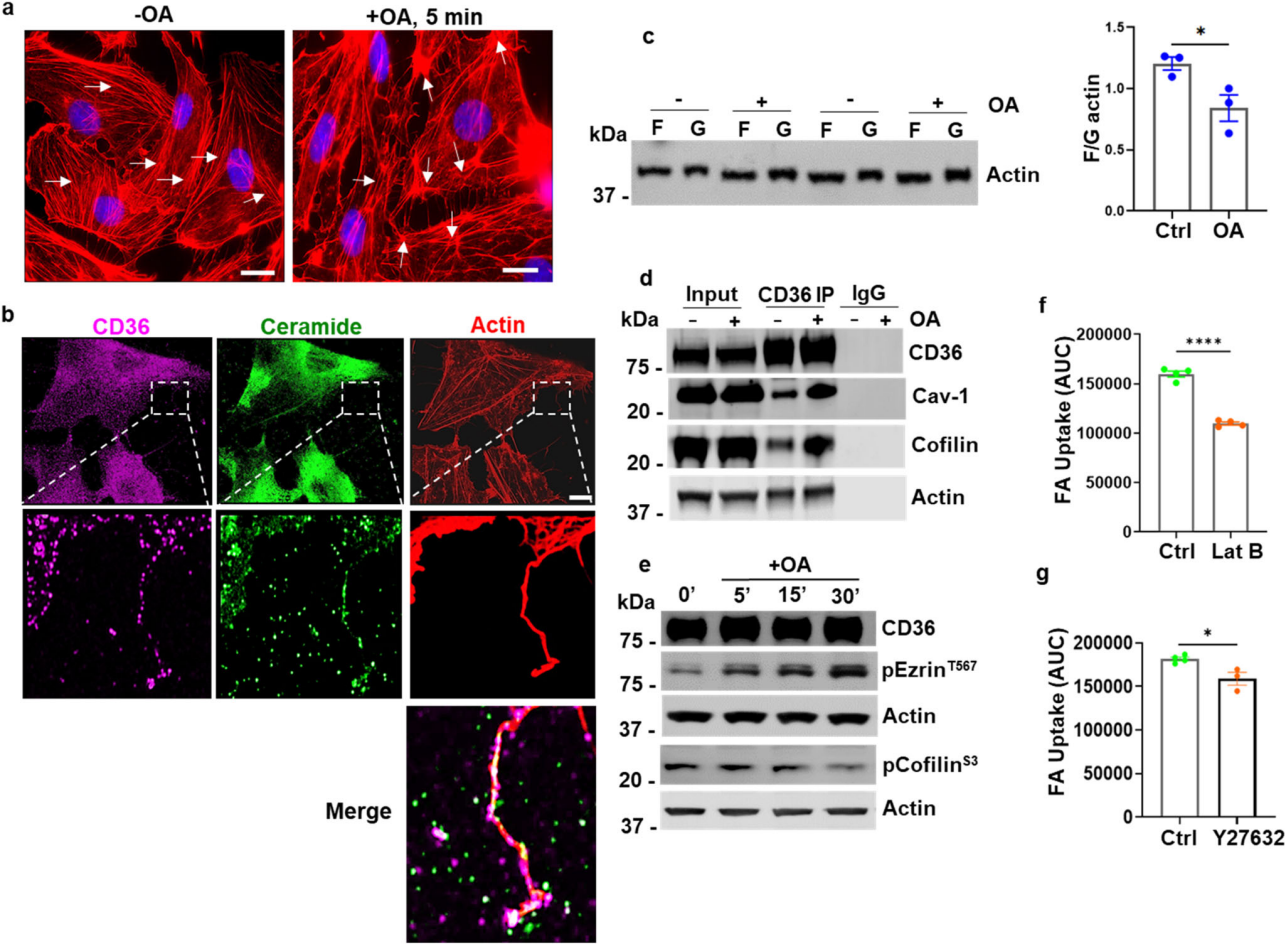
Oleic acid induces rapid actin remodeling in MECs. **a** Changes in actin stress fibers in cells exposed to OA: hMECs, were treated with oleic acid (OA:BSA, 100 µM:50 µM) for 5 min, fixed and F-actin visualized with TRITC-Phalloidin. Scale bars: 10 µm. **b** OA induces cellular actin extensions. hMECs processed as above were immunostained for CD36, ceramide, and F-actin. Lower panel magnification shows cellular extensions containing actin that are decorated with ceramide and CD36-positive sEVs, as shown in the merge. Scale bar: 10 µm. **c** Reduced F/G actin ratio in OA-treated hMECs: Samples from G- and F-actin fractionation of cells treated as in (**a**), resolved on SDS-PAGE and immunoblotted for β-actin, quantified in adjoining graph. *n* = 3 independent experiments, *p* < 0.05 by unpaired t test. **d** Enhanced interaction of CD36 with cofilin, actin, and Cav-1 in OA-treated hMECs: Cells were treated with OA:BSA, 100 µM:50 µM, for 30 min. CD36 IP from equal lysate protein (input) were immunoblotted and probed for CD36, Cav-1, cofilin, and actin. **e** Altered phosphorylation of actin-binding proteins ezrin (T567) and cofilin (S3) after OA addition. **f**, **g** Actin remodeling is critical for FA uptake. FA uptake (AUC) in cells treated with actin disrupting Latrunculin B (**f**) versus the ROCK1/2 inhibitor (Y27632) (**g**). *n* = 4 experiments for (**f**), ****p < 0.0001 by unpaired t test. *n* = 3–4 independent experiments for (**g**), *p < 0.05 by unpaired t test. All data are means +/− SEM.

Since endocytosis and vesicular traffic depend on the cytoskeleton^74^, we next examined if rearrangement of cellular actin is required for OA uptake by MECs. OA addition was found to rapidly alter morphology of actin stress fibers in hMECs causing peripheral fiber clustering close to actin-containing cellular extensions (Fig. 7a, arrows). The extensions, which protruded into the extracellular space between cells, appeared to be decorated with sEVs (Fig. 7b, insets). Actin-containing extensions can help transfer sEVs to recipient cells^75,76^. Actin remodeling in response to OA was demonstrated by a change in ratio of filamentous (F) to monomeric globular (G) actin (Fig. 7c, *p* *< 0.05, *n* = 3 experiments) and by increased CD36 association with actin and cofilin, a key protein that depolymerizes actin and maintains a dynamic F/G actin equilibrium (Fig. 7d). In addition, OA induced ezrin phosphorylation at activatory threonine 567 while it induced dephosphorylation of cofilin at inhibitory serine 3 (Fig. 7e), both changes would facilitate actin remodeling. To directly test requirement of actin remodeling in FA uptake we examined the effect of latrunculin B, which binds actin monomers preventing them from polymerizing thereby affecting G-actin recycling^77,78^. Pretreatment of hMECs with latrunculin resulted in substantial reduction of FA uptake (Fig. 7f, *p* < 0.0001, *n* = 4 experiments). In contrast, FA uptake was only slightly suppressed by Y-27632 which inhibits the Rho GTPase effectors ROCK1 and 2 (Rho-associated coiled-coil-containing protein kinase) that regulate actin organization for cell motility and other cellular functions^79^ (Fig. 7g, *p* < 0.05, *n* = 4 ctrl, 3 Y-27632). In line with relevance of actin remodeling in facilitation of CD36-mediated FA uptake, CD36 knockdown in hMECs altered expression of proteins implicated in actin organization such as fascin, profilin, myosin light chain kinase (MLCK), coronin7 and fibrillin 2 (Supplementary Tables 1 and 2).

### Endothelial cells rapidly transfer FA-sEVs to myotubes

**Fig. 8:**
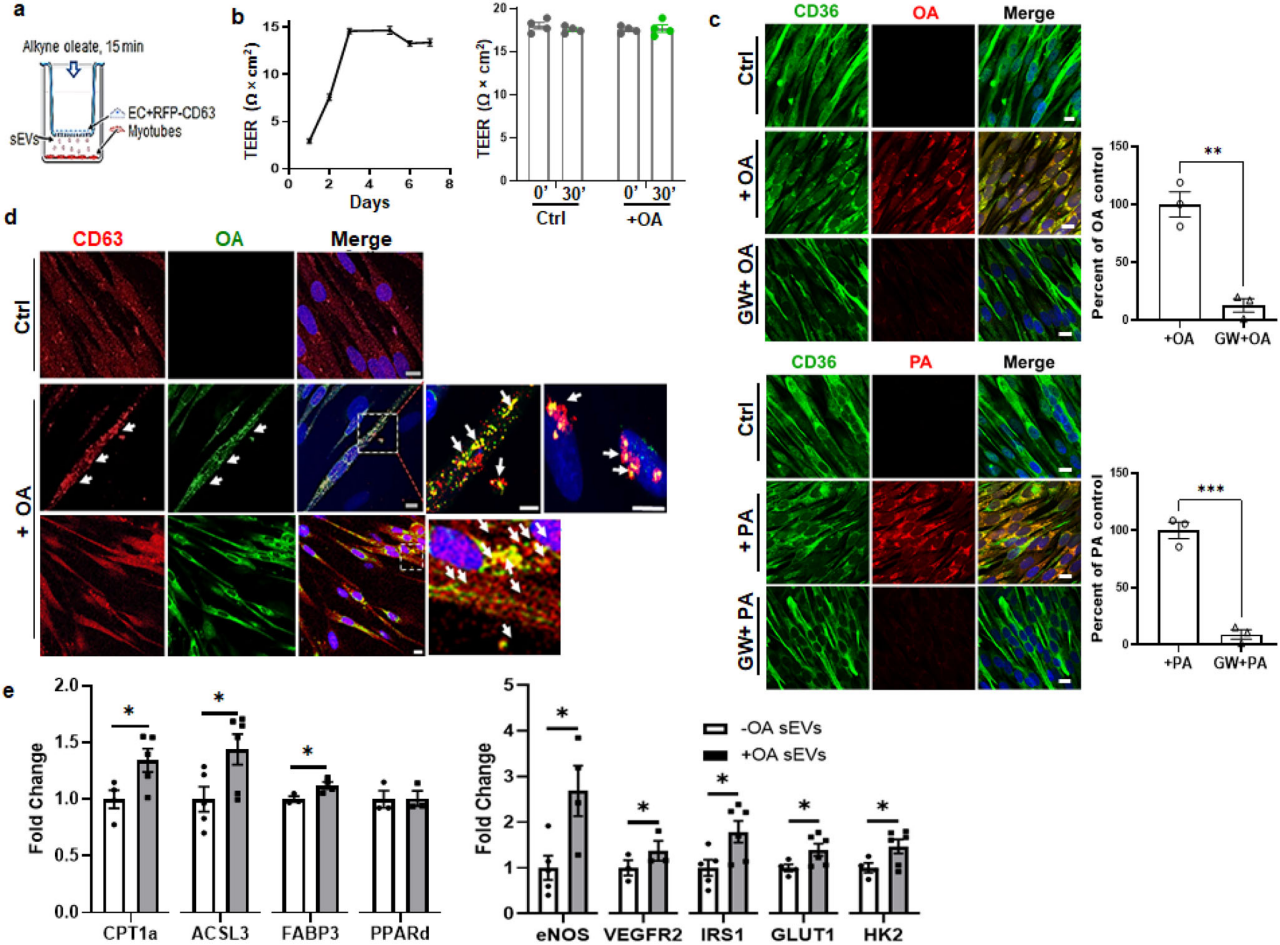
Endothelial cells transfer FA to myotubes via sEVs. **a** Cartoon depicting the transwell setup. ECs were grown on filters to a confluent monolayer in the upper chamber while myotubes were grown in the lower chamber. Akyne FA was added to the upper chamber. FA transfer to myotubes was visualized by fixing the cells and performing Click chemistry. **b** Barrier integrity of hMECs grown on transwells until 3 days post confluence. Barrier integrity was assessed using TEER measurement. The TEER plateau indicates that the barrier is fully formed (left panel, *n* = 3 experiments). The TEER plateau was unaffected by OA addition, right panel. *n* = 4 independent experiments, *p* > 0.05 by unpaired t test. Data are means +/− SEM. **c** hMEC transfer OA and PA to myotubes through sEVs. Human myoblasts were grown on coverslips and differentiated into myotubes. The hMECs were grown on filters in transwells until 3–4 days post confluence. The myotubes on coverslips were placed in the bottom wells of the transwells. **c** Alkyne OA (upper panels) or PA (lower panels) was added (25 µM, 30 min) in presence or absence of the nSMase2 inhibitor GW4869 (GW, 10 µM, 10 min). Myotubes were clicked and processed for CD36 immunostaining and confocal microscopy. The percent GW4869 inhibition of OA and PA flux to myotubes is shown in adjacent graphs. *n* = 3 independent experiments, **p < 0.01 for OA, ***p < 0.001 for PA by unpaired t test. Data are means +/− SEM. Scale bar: 10 µm. **d** Alkyne OA transfers with the exosome marker CD63 when hMECs express RFP-CD63 (red). Alkyne OA was added to the hMECs and 30 min later myotubes were processed to visualize OA and CD63. Top control panel: Myotubes under control hMEC (no OA added) showed low diffuse red CD63 signal. Panels + OA: Myotubes cultured below OA-treated hMECs showed bright intracellular red CD63 puncta that were also positive for OA (green azide Alexa Fluor-488). Note green OA signal was used to distinguish it from the red RFP-CD63 In the middle +OA panel, arrows point to puncta positive for CD63 and OA. Inset: Myotubes at higher magnification showing OA/CD63 vesicles inside and outside the cell (arrows) and an additional higher magnification highlights OA/CD63 vesicles with perinuclear localization. Bottom panel shows an experiment similar to that in the middle panel to further illustrate the yellow merge of hMEC-transferred OA (green) with CD63 (red) inside myotubes. Scale bars: 10 µm. **e** hMEC FA-sEVs alter gene expression in myotubes. qPCR analysis of myotubes treated 2 h with sEVs from ECs with and without OA treatment. *CPT1a*: carnitine palmitoyl transferase a, *ACSL3*: Acyl-CoA synthase long chain 3, *FABP3*: FA binding protein 3, *PPARδ*: Peroxisome proliferator activating receptor delta, *eNOS*: endothelial nitric oxide synthase, *VEGFR2*: Vascular endothelial growth factor receptor 2, *IRS1*: Insulin receptor substrate 1, *Glut1*: Glucose transporter 1, *HK2*: hexokinase 2. *n* = 3–6 independent preparations, *p < 0.05 by unpaired t test. Data are means +/− SEM.

Transfer of sEVs from MECs for uptake by myotubes was visualized in a polarized setting with hMECs cultured in the upper chamber of a transwell, and myotubes placed underneath in the lower chamber (Fig. 8a). Barrier integrity of the MEC monolayer was assessed by trans-endothelial electric resistance (TEER), which measures monolayer impermeability based on resistance to ionic flux between cells (paracellular)^80^. As the MECs seeded on the filter increased in number, and intercellular junctions started closing the gaps between cells, electric resistance increased, reflecting the progressive decrease in monolayer permeability, as is shown in Fig. 8b left panel. TEER reached a plateau (maximum resistance) when the barrier was fully formed. The TEER plateau was not altered by OA addition indicating that OA did not breach the barrier (Fig. 8b right panel). Further confirmation of barrier integrity used C12 Bodipy FA (red) and Dextran-FITC (green) added to the upper chamber in absence or presence of OA. Fluorescence of both was monitored in the lower chamber. Dextran transfer shown as percent of input in the top compartment was very low, around 0.02% at 30 min which is our assay time, and it remained low (0.2% of input) at 240 min indicating stable monolayer integrity over time. Transfer of both dextran and Bodipy was unaffected by added OA (Supplementary Fig. 4a).

The hMECs were transduced to express the red fluorescent CD63 (RFP-CD63), a plasma membrane protein and sEV marker, before culture on the transwell filters until 3–5 days post confluence. There was low transfer of CD63 to myotubes during a 30 min basal period as deduced from the diffuse red signal in Supplementary Fig. 4b. OA addition to hMECs in the upper chamber resulted in detectable red CD63 transfer from hMECs to myotubes reflecting sEV uptake (Supplementary Fig. 4b, middle and bottom panels) and the red CD63 signal merged with green CD36 immunostaining (Supplementary Fig. 4b, middle and bottom panels) consistent with transfer of EC CD36 in CD63 sEVs.

We confirmed in transwells that the sEV transfer mechanism applies to PA and OA by adding alkyne OA or PA to hMECs on filters in the top chamber, then evaluating transfer to myotubes in the bottom well. Red signal from OA (Fig. 8c, upper panels, middle lane) and PA (Fig. 8c, lower panels, middle lane) was measured in myotubes and co-localized with CD36 (Fig. 8c middle lanes, merged images). Importantly, this transfer was blunted, becoming almost undetectable when the hMECs were treated with GW4869, a neutral sphingomyelinase 2 (nSMase2) inhibitor, which blocks sEV generation. The inhibition observed was comparable for OA and PA (Fig. 8c adjacent graphs, *n* = 3, *p* < 0.01 for OA and *p* < 0.001 for PA). These results showed that the FA (OA or PA) transfers to myotubes in sEVs and blocking sEV generation markedly suppresses the transfer. These results also indicate that the major FA transfer pathway is across EC through transcytosis and sEV generation, as the monolayer is impermeable to paracellular FA flux. We next visualized OA transfer from hMECs to myotubes in labeled sEVs. We performed the transwell studies with alkyne OA added to upper chamber hMECs expressing the RFP labeled exosome marker CD63. The OA recovered in lower chamber myotubes was clicked with fluor-azide as before. In this experiment, unlike in Fig. 8c, OA was visualized with Alexa fluor-488 (green) instead of fluor-555 to distinguish it from the red RFP-CD63. During a 30 min basal period (no OA added) little RFP-CD63 transfer to myotubes was seen (Fig. 8d, top panel). OA addition (Fig. 8d, middle and bottom panels) stimulated transfer of RFP-CD63 (red) and OA (green) to myotubes, where OA and CD63 co-localized forming intracellular yellow puncta, as highlighted in the two insets of the middle panel and in the inset of the bottom panel. These data supported FA transfer from hMECs to myotubes in RFP-CD63 sEVs. Taken together, these data show that FAs are delivered by ECs to parenchymal cells through generation of sEVs.

The sEVs are critical in cell-cell communication and metabolic regulation^70,81–83^. We examined whether exposure to sEVs from FA-treated ECs alters expression of key regulatory proteins in myotubes. Incubating myotubes for 2 h with sEVs from OA-treated hMECs (Fig. 8e) significantly increased expression of key genes of FA metabolism, as compared to sEVs from untreated hMECs; carnitine palmitoyl transferase 1a (*CPT1a*), acyl-CoA synthetase long chain 3 (*ACSL3*), fatty acid binding protein 3 (*FABP3*), and of those for glucose utilization; glucose transporter 1 (*GLUT1*), insulin receptor substrate 1 (*IRS1*), and hexokinase 2 (*HK2*). Expression of the vascular regulator endothelial nitric oxide synthase (*eNOS*) increased and that of vascular endothelial growth factor receptor 2 (*VEGF-R2*). Thus, sEVs from FA-treated hMECs increased myotube expression of key metabolic and vascular genes.

### The sEV pathway contributes to tissue FA uptake and regulation of blood FAs

**Fig. 9:**
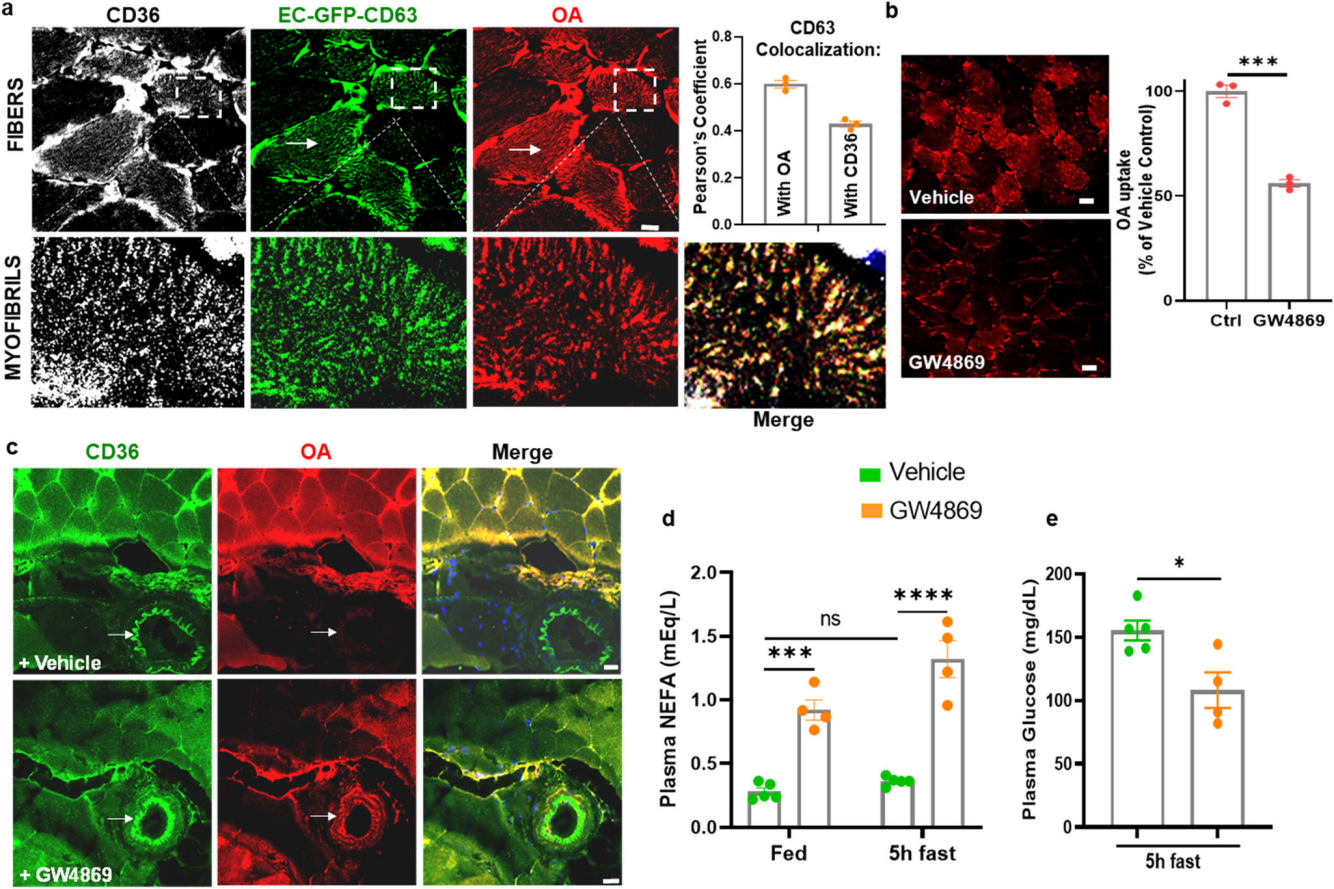
EC secreted sEVs transfer FAs to muscle in vivo. **a** Visualization of OA uptake in reporter mice with EC-expression of emGFP-CD63. Mice were given alkyne OA (25 µM) retro-orbitally, euthanized 30 min later, and skeletal muscle fixed, clicked, and immunostained for CD36. Top panel shows cross sections of muscle fibers with arrows pointing to myofibrils inside the fibers. Higher magnification insets in the bottom panel show association of CD36/OA/CD63 staining with the myofibrils inside fibers. Adjoining graph shows Pearson’s co-localization coefficient for CD63/OA and CD36/CD63 (*n* = 3 animals, 8–10 individual muscle fibers/animal). **b** OA uptake by muscle fibers is suppressed by GW4869. Mice were injected with vehicle (DMSO) or GW4869 (2.5 µg/g intraperitoneally for 4 days) then given alkyne OA (25uM) retro-orbitally. GW4869 reduced OA uptake by muscle fibers as quantified in adjoining graph. *n* = 3 mice per group for vehicle and GW4869 treatment, 10–15 individual muscle fibers/mouse ***p < 0.001, unpaired t test. Data are means +/− SEM. Scale bar, 10 µm. **c** GW4869 induced reduction in OA uptake by muscle fibers associates with trapping of the OA in blood vessels. Skeletal muscle from a vehicle-treated mouse (upper panel) shows strong OA uptake into fibers and OA absence from a nearby CD36-expressing vessel (arrows). Muscle from a GW4869-treated mouse (lower panel) showed reduced uptake into fibers, and OA retention in a nearby CD36-expressing vessel (arrows) can be seen. Scale bar 20 µm. **d** GW4869 increases blood levels of non-esterified FAs (NEFAs). Mice were treated with GW4869 or vehicle, as in (**c**) at end of the dark period (fed) or after a 5 h fast. NEFAs were measured in tail vein blood. *n* = 4–5 mice/group, ****p < 0.0001, ***p = 0.0002 by two-way ANOVA adjusted for multiple comparisons. Data are means +/− SEM. **e** GW4869 reduces blood glucose. Mice (vehicle or GW4869 treated) were fasted for 5 h and blood glucose measured in tail vein blood. *p < 0.05 by unpaired t test, data representative of two cohorts *n* = 4–5 mice per/group. Data are means +/− SEM. Note: Blood glucose in C57bl6 mice (fed or fasted) are normally higher as compared to levels in other commonly used strains^109, 110^.

We next examined in vivo operation of the sEV-exosome pathway in FA uptake. We used a reporter mouse with EC-specific expression of emeraldGFP-tagged CD63 (emGFP-CD63)^84^ to visualize transfer of FA-exosomes from ECs to muscle fibers. Alkyne OA was given retro-orbitally to the mouse and 30 min later, hindlimb plantar flexor muscles were dissected. Frozen sections (10 µm) were fixed, processed for FA click reaction, and immunostained for CD36. Confocal images of muscle fibers (Fig. 9a, top panel) showed expression of CD36 (white), EC-emGFP-CD63 (green), and OA (red) at the periphery of muscle fibers as well as on myofibrils inside muscle fibers which appeared as puncta inside the fibers. Previously, lipid droplets and mitochondria were shown to associate with the myofibrils of muscle fibers^85^. High-magnification images to highlight the myofibrils (Fig. 9a, bottom panel) were used to quantify co-localization of OA and CD63 inside the fibers. This yielded a strong Pearson coefficient of 0.6 consistent with OA traveling in CD63 labeled sEVs to myotubes (Fig. 9A, adjoining graph *n* = 3 animals, 8–10 muscle fibers/animal). The co-localization coefficient of CD36 and emGFP-CD63 (0.43) was lower, likely reflecting immunostaining of both endogenous muscle CD36 and the CD36 transferred from ECs.

We tested importance of the sEV pathway in muscle uptake of circulating FAs by giving wild-type mice the nSMase2 inhibitor GW4869 (intraperitoneal, 4 days) and then a retro-orbital injection of alkyne OA. Muscles were excised 30 min after the OA, sectioned, fixed, clicked, and stained as before. Confocal microscopy showed that GW4869 reduced by about 50% OA staining of muscle fibers (Fig. 9a, adjoining graph, *p* < 0.001, *n* = 3 animals per group, 10–15 muscle fibers/animal).

Muscle uptake of circulating OA was paralleled with OA depletion from blood vessels (Fig. 9c arrows). Muscle fibers of vehicle-treated WT mice given alkyne OA stained brightly for CD36 (green) and OA (red) while a nearby blood vessel expressing CD36 (Fig. 9c, top panel, arrows) had little red OA staining indicating OA had already transferred out of the vessel. The opposite was seen in muscle of GW4869-treated mice where fibers had low OA staining while a nearby blood vessel expressing CD36 retained substantial OA signal (Fig. 9c lower panel, and Supplementary Fig. 6a, lower panel).

To confirm that inhibition of exosome biogenesis in vivo causes endothelial retention of circulating FA, we measured plasma-free FA (NEFA) in mice given GW4869. We found (Fig. 9d) that these mice had increased blood FAs when tested fed or after a 5 h fast (Fed: vehicle vs GW4869 *p* = 0.0001; Fasted: vehicle vs GW4869 *p* = 0.0069, *n* = 4–5 mice, two-way ANOVA with adjustment for multiple comparisons). Interestingly, GW4869 reduced blood glucose in mice, consistent with the reduced muscle FA uptake (Fig. 9e, *p* < 0.05, *n* = 4–5 mice). The effects of GW4869 on blood FAs and glucose mimic effects observed in *Cd36*^*−/−*^ and *EC-Cd36*^*−/−*^ mice^9,86^. In line with this, GW4869 treatment did not alter FA or glucose levels in *Cd36*^*−/−*^ mice (Supplementary Fig. 6b). We also examined effect of in vivo inhibition of Src by Scr inhibitor-1 on alkyne PA uptake. Src-1 blocks Cav-1^Y14^ phosphorylation and caveolae endocytosis, without eliminating ceramide formation (shown earlier in Fig. 6e, f). Animals injected with Src-1 showed reduced alkyne PA uptake in muscle fibers as compared to vehicle-treated mice. Src inhibition also associated with more retention of PA in blood vessels, similar to alkyne OA vessel retention in GW4869-treated mice (Supplementary Fig. 6c, d). Injection of Src-1 also significantly raised blood FAs (Supplementary Fig. 6e, *p* < 0.01, *n* = 5–6 mice per group) Together these data support the physiological role of the sEV pathway in endothelial transfer of circulating FAs to underlying tissue cells.

## Discussion

Since its identification as a FA transporter, numerous studies have documented role of CD36 in tissue FA uptake and utilization in mice and humans, as recently reviewed^12,13,87^. Endothelial cell-specific deletion of CD36 in mice showed EC CD36 is required for tissue FA uptake and recapitulated many metabolic effects of global CD36 deficiency^9^. This study documents that ECs transfer FAs from the circulation to underlying tissue through sEVs. Blocking sEV formation increases blood FA levels and replicates phenotypes found with CD36 deletion.

**Fig. 10:**
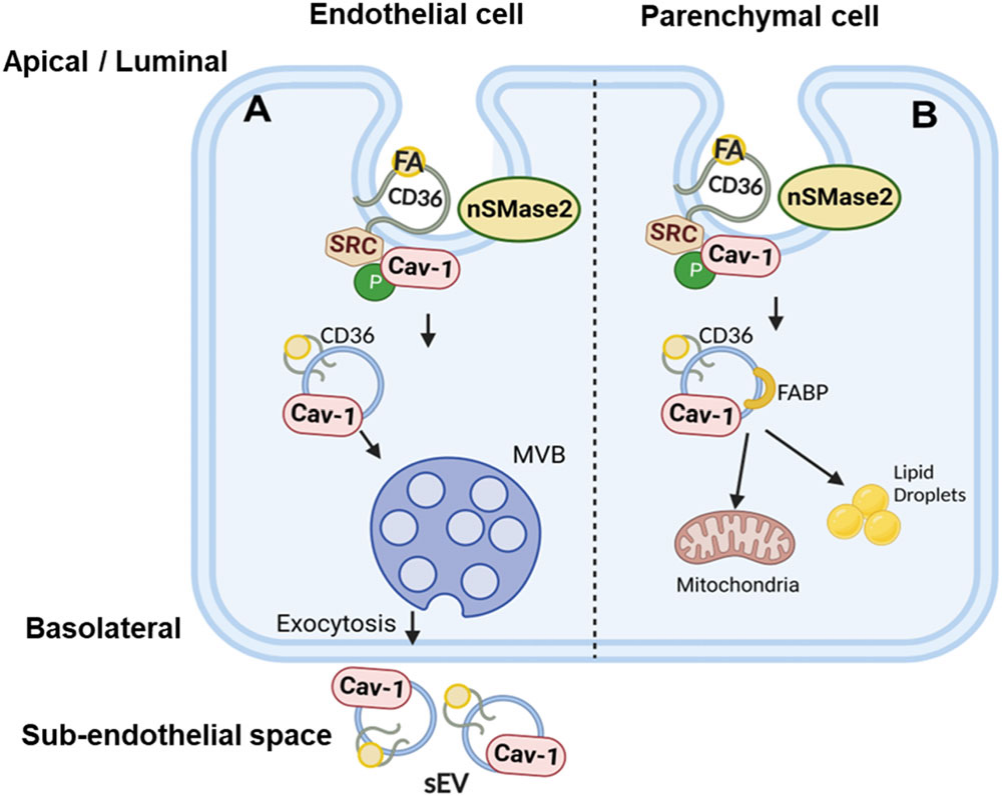
Schematic of the EC FA export pathway. **A** The polarized ECs control tissue FA uptake by transcytosis of circulating FAs and their export in small extracellular vesicles (sEVs) to parenchymal cells. FA binding to CD36 recruits Src to phosphorylate Cav-1 at tyrosine14, which disrupts the caveolae coat. Associated activation of nSMase2 generates the destabilizing ceramides in the caveolae. Both events initiate caveolae fission from the membrane. The caveolae internalize into small ceramide-rich intracellular vesicles (IVs) that are then sorted into the lumen of multivesicular bodies (MVBs). The MVBs fuse with the basolateral membrane to release exosome-like small sEVs in the sub-endothelial space. The sEVs export FA and other cargo to parenchymal cells. **B** Possible alternate fate of endocytosed vesicles in parenchymal cells. The IVs generated in response to FA are likely to be heterogenous and a subset possibly less enriched in specific ceramides might during traffic or sorting associate with proteins such as FABPs that help target the IVs to cellular organelles instead of for secretion as sEVs. This latter fate might predominate in myocytes, cardiomyocytes, and adipocytes where most FAs are kept for local use or storage.

We described how FA interaction with CD36 in the apical membrane of ECs triggers CD36/Src coordinated FA internalization through caveolae budding into intracellular vesicles (IVs) that travel and are secreted as sEVs at the basolateral side. We used a combination of approaches including microscopy, biochemical analysis, and real-time FA uptake assays to identify the transcytosis pathway. We visualized FA internalization by CD36 and Cav-1 using confocal microscopy coupled with click chemistry of alkyne OA or PA. Using TEM with nanogold PA, we highlighted FA endocytosis into caveolae-derived intracellular vesicles (IVs), sorting of the FA-IVs into MVBs, and the secretion of sEVs that contain FA, CD36, and Cav-1. Secretion of these sEVs was also shown by confocal microscopy. The isolated sEVs have common exosome markers and a diameter between 80–100 nm consistent with their caveolae origin. Three critical mechanisms were identified in EC CD36-mediated transcytosis of FAs: First, FA-induced Src phosphorylation of residue Y14 in Cav-1. Both Src inhibition and the expression in MECs of Y14 mutated Cav-1 were shown to suppress FA uptake by confocal microscopy and real-time FA uptake. Second, FA-induced generation of ceramides by membrane sphingomyelinase. Inhibition of membrane nSMase2 suppressed FA-induced ceramide formation, FA internalization, and FA uptake by MECs. Third, FA-induced rearrangement of cellular actin. The FAs triggered phosphorylation of actin remodeling proteins cofilin and ezrin and enhanced CD36 association with cofilin and actin. Inhibition of actin remodeling reduced FA uptake. The above data documented the details of CD36 facilitated EC FA transcytosis and secretion in sEVs as highlighted in the schematic model of Fig. 10.

Both FA-treated and untreated ECs, and ECs with or without CD36 expression released exosome-like sEVs with a diameter between 80–100 nm. This is consistent with ECs releasing sEVs at a continuous low basal rate while addition of FAs rapidly upregulates caveolae endocytosis and sEV formation. Due to the basal output of sEVs and the FA-containing sEVs being a subset of the total sEVs secreted, our lipidomics measurements represent the total sEV pool. However, despite this limitation, conclusions can be made related to the changes induced by OA treatment in WT versus CD36^−/−^ cells. The percent OA in sEVs doubled with FA treatment when ECs expressed CD36 but not when ECs lacked CD36. A couple of observations suggested that FA addition disrupts distribution of specific, shape-inducing lipids, known to impact caveolae stability^61^. We measured opposite OA-induced changes in sEV ceramide Cer 18:1;O2/24:1, which increased in sEVs from WT ECs versus decreased in sEVs from CD36^−/−^ ECs (Fig. 5d). Lysophosphatidylcholine (LPC) also showed opposite OA-induced changes, decreasing in sEVs from WT ECs versus increasing in sEVs from CD36^−/−^ ECs. Thus, OA increased Cer 18:1;O2/24:1 and decreased LPC in sEVs from cells that endocytosed the caveolae with FA and the opposite occurred in sEVs from ECs where caveolae/FA endocytosis was suppressed. Effects on caveolae lipids might have contributed to the role of Src and CD36 in FA transcytosis. Src phosphorylation of Cav-1^Y14^ alters Cav-1 interactions with other caveolae resident proteins, and many of these proteins bind and stabilize caveolae lipids^88^. In the case of CD36, in addition to its interaction with FA and association with Src, it might exert regulation through its binding phosphatidylserine^89^, which is enriched in caveolae and important for activation of nSMase2 and ceramide generation^68^. Additional studies are needed to examine how the FA-CD36 interaction alters caveolae lipid distribution as it relates to caveolae endocytosis and sEV secretion.

Physiological relevance of the FA-sEV pathway was shown with transwells where inhibition of the sEV pathway by GW4869 markedly suppressed FA flux from upper chamber ECs to myotubes in the lower chamber. Also upper chamber hMECs expressing the exosome marker RFP-CD63 transferred OA together with CD63 to myotubes. In addition, a mouse model with endothelial-specific expression of emGFP-CD63, click chemistry of intravenously injected alkyne OA visualized OA transfer with emGFP-CD63 to muscle fibers. Inhibition of exosome formation suppressed muscle fiber FA uptake in vivo, increased blood FAs, and reduced glucose levels but only when mice expressed CD36. Src inhibition to block Cav-1 phosphorylation also reduced FA uptake by muscle and increased blood FAs in mice. Our studies suggest that the sEV pathway plays an important physiological role in uptake of circulating FA. Additional work should further assess its quantitative contribution, particularly under different metabolic states. In this context, a previous report noted that in mice circulating exosomes have more CD36 postprandially as compared to after an overnight fast, and CD36 acquisition was important for loading BODIPY-FA into exosomes. Injection of mice with exosomes prelabeled with BODIPY-FA resulted 1.5 h later in recovery of fluorescence in the heart^90^. The origin of the postprandial CD36 expressing exosomes was not determined but might reflect in part spillover into the circulation of EC-derived FA-sEVs generated during intravascular lipolysis. How the EC FA-sEV pathway is affected by feeding, overfeeding or high fat intake, and the metabolic consequences remain to be elucidated.

The FA sEV pathway will be relevant to our understanding of how circulating FAs influence tissue crosstalk and metabolic health^4,5^ and how in some cases FAs can promote metabolic disease or cancer metastasis^12,91,92^. Exosomes are critical in cell-cell communication and influence the phenotype of recipient cells by carrying microRNAs, bioactive lipids, and other regulatory factors. The FA-treated ECs generated sEVs that enhanced expression of key metabolic enzymes of both FA and glucose in myotubes. These data could help explain the reported effects of FA supply in inducing adaptations of skeletal muscle FA uptake and oxidation^93^. The sEVs also increased expression in myotubes of eNOS and that of VEGF-R2 suggesting exosomes contribute to vascular regulation and capillary maintenance. In human muscle, a 3-h bout of exercise increases gene expression of VEGF-R2, eNOS, and HK2, effects referred to as hormetic, meaning adaptive and aimed at reestablishing homeostasis^94^.

During their biogenesis, intracellular traffic, and secretion, the sEVs integrate various proteins such as membrane transporters, tetraspanins, enzymes, and proteins important for membrane fusion or for targeting sEVs to specific cells or cellular organelles, etc^95^. The cargo of the FA-sEVs likely includes proteins other than CD36 that function in FA uptake, e.g., FA acyl-CoA synthases^96^ and cytosolic FA binding proteins, FABPs^97,98^. These proteins would serve as intracellular docking sites targeting the vesicles to specific cellular organelles. The FA-sEV pathway is likely to predominate in the polarized ECs. These cells control tissue FA uptake but utilize little FAs, and as we showed, transcytose the FAs for export in sEVs to parenchymal cells. However, in contrast to ECs, parenchymal cells keep most FAs for local use (e.g., cardiomyocytes, myocytes, adipocytes). In these cells, the FA-sEV which might associate with FABPs during traffic or sorting would be primarily directed to cellular organelles for processing, e.g., to lipid droplets^50^, mitochondria, or the nucleus, as highlighted in Fig. 10. This might explain the marked defect in lipid droplet formation we previously observed in the heart of mice with cardiomyocyte CD36 deletion despite presence of normal myocardial FA uptake. This observation contrasted with the findings in mice with EC CD36 deletion where uptake and lipid formation were both suppressed^9^.

Vesicular recycling of CD36 between endosomes and sarcolemma regulates cell surface CD36 and myocardial FA uptake^99^ and is governed by assembly of the vacuolar H^+^-ATPase (V-ATPase) complex which acidifies endosomes. Endosome alkalinization pursuant to V-ATPase disassembly by FAs was reported to induce permanent CD36 relocation to the membrane with dysfunction of FA uptake^87^. Interestingly, V-ATPase also regulates the sEV pathway and a decrease in its activity promotes sEV secretion by reducing MVB acidification^69,100^. Regulation of V-ATPase in different cellular organelles could play an important role in directing the endocytosed FA vesicles. We speculate that some of the FA handling proteins, notably the FABPs will associate with the FA-EVs during their sorting and play a role in directing the vesicles in a cell type-specific manner, to lipid droplets, mitochondria, nuclei, or other sites.

The sEV FA uptake pathway might contribute to some of CD36 actions in metabolism, atherosclerosis, and immunity^11,91,101^ as the findings could apply to CD36 ligands other than FAs. These could include native or oxidized lipoproteins, the multifunctional integrins, amyloid beta, pathogens^20,56^ etc., and could have relevance to other CD36 actions^11,91,101^. Our findings connect FA and ceramides independent of ceramide synthesis as they show that FAs regulate membrane ceramide metabolism, which in turn regulates FA uptake. It will be important to understand the implications of this new connection in the etiology of FA- or ceramide-induced cellular pathophysiology^102^.

## Methods

### Mice

C57BL/6 *Cd36*^*−/−*^ mice^27,103^ and wild-type (WT) controls were used for isolation of lung microvascular cells. A mouse with EC-specific expression of emGFP tagged CD63, an exosome marker, was generated by crossing CD63-emGFP(loxP/stop/loxP) with Cdh5-Cre(ERT2) mice^104^ to generate tamoxifen inducible EC-CD63(emGFP +) mice as previously described^84^. All experiments included male and female animals. The mice were maintained under standard laboratory conditions with a 12-h light/12-h dark cycle. The room temperature was maintained between 20 and 23 °C, and the humidity ranged from 30 to 70%. The mice were provided with rodent chow and water throughout the study period unless indicated. Mice were euthanized using CO_2_ inhalation according to institutional guidelines. Protocols were approved by the Washington University Institutional Animal Care and Use Committee and complied with ethical regulations for laboratory animal studies.

### Cell isolation, culture, and treatment

Primary mouse microvascular ECs (mMECs) and human dermal microvascular ECs (hMECs) were used. Mouse MECs were isolated from lungs of WT and *Cd36*^*−/−*^ mice after phosphate-buffered saline (PBS) perfusion. The lungs were enzymatically digested (collagenase I, Gibco) and the separated cells (GentleMACS separator, Miltenyi Biotech) pelleted and resuspended in PEB buffer (1x PBS, 2 mM EDTA, 0.5% BSA). After addition of FcR blocking reagent (Miltenyi Biotech) the cells were incubated (20 min, 4 °C) with CD31-conjugated microbeads and the suspension passed through a separation column (MACS, Miltenyi Biotech) followed by column washing (3X, PEB), cell elution and plating in endothelial growth media (EGM2-MV, Lonza) either on gelatin-coated coverslips, or in 6-well plates. The mMECs were only passaged twice. Human MECs (Lonza) were cultured in endothelial media (EGM-2 MV, Lonza) and passaged less than 5 times. To knockdown CD36, hMECs were transfected with control siRNA or CD36 siRNA (4392420, assay ID: s2646, ThermoFisher Scientific) using Lipofectamine RNAiMAX (ThermoFisher Scientific). For CD36 overexpression, mEmerald-CD36 (Addgene, #54030) and mCherry-Cav-1 (Addgene, #55008) were transfected with Lipofectamaine LTX (ThermoFisher Scientific). The dominant-negative Cav-1^Y14F^ was generated by site-directed mutagenesis (Quikchange XL, Agilent).

To inhibit neutral sphingomyelinase 2 (nSMase2), serum-starved MECs were pretreated with GW4869 (10 µM, 10 min, Sigma). To inhibit Src kinase, Src inhibitor-1 (S2075, Millipore Sigma) was added (10 µM, 2 h) to hMECs and mMECs in the second half of the 4 h serum starvation. To check specificity of ceramide antibody, hMEC were serum starved for 4 h and treated with bacterial Smase (25 miliunits,10 min) either without or followed by ceramidase (20 mUnits, 10 min)^60^. Cells were fixed, permeabilized, and processed for immunofluorescence. Mouse monoclonal ceramide primary antibody was used followed by goat anti-mouse antibody conjugated to Alexa Fluor 488. F-actin and nuclei visualized with TRITC-Phalloidin and DAPI, respectively.

### Cell fixation, immunostaining, and FA click chemistry

Alkyne FAs (Cayman Chemical) and click chemistry^35^ were used to visualize FA uptake. Serum-starved (4 h) primary mMECs or hMECs grown on coverslips were treated with alkyne FAs added with or without bovine serum albumin (BSA, 0.2%) at the indicated concentrations (10–15 µM) and times (10–20 min). Cells were rinsed (PBS, 2% FBS), fixed in 4% paraformaldehyde (PFA), washed (PBS), permeabilized (0.05% Triton X-100, 10 min), and washed (PBS, 2% FBS). Reaction with Alexa Fluor 488, 555, or 568-azide to label the alkyne FA used a Click-iT Cell Reaction Buffer Kit (ThermoFisher Scientific). Cells were gently washed (PBS, 2% FBS) and immunostained as needed.

Microvascular cells, mMECs or hMECs on coverslips or in 6-well plates were treated with native oleic acid (OA) complexed with FA-free bovine serum albumin (OA-BSA, 100 µM:50 µM) for indicated times. Cells were fixed with 4% PFA, permeabilized with 0.05% Triton X-100, washed (PBS), blocked (1 h, 2% donkey serum in PBS), and incubated with primary antibodies overnight at 4 °C. After 3 PBS washes, cells reacted 1 h with Alexa fluorophore secondary antibodies (ThermoFisher Scientific) were washed and mounted in gelvatol. Image acquisition and processing used, respectively, a Zeiss LSM 880 Airyscan Confocal Microscope (×40 or ×63 objective) and Zeiss Zen (Blue) software.

### In vivo inhibition of nSMase2 and FA click chemistry

Wild-type and *Cd36*^*−/−*^ mice were intraperitoneally (IP) injected either with DMSO (vehicle control) or with 2.5 µg/g GW4869^105^, an inhibitor of nSMase2 for 4 days (*n* = 4–5/genotype/group). Src inhibitor-1 or vehicle were IP injected (2 mg/kg) for 4 days (*n* = 4–5/genotype/group)^106^. Mice were either studied fed (at the end of the dark period) or after a 5 h fast. Blood was collected from the tail vein and non-esterified FAs (NEFA) were measured (Wako Diagnostics, USA). For in vivo click chemistry, 5 h fasted mice (DMSO or GW4869 groups) were retro-orbitally injected under 2% isoflurane 100 µl of warmed PBS with alkyne OA (to yield ~25 µM OA in the circulation based on 2 mL blood volume in a 25–30 g mouse). After 30 min, mice were euthanized by cervical dislocation under 2% isoflurane and skeletal muscle processed for vapor fixation. Plantar flexor muscles were dissected from the hindlimb, frozen in liquid nitrogen cooled isopentane, and stored at −80 °C until sectioning. Sections, 10 µm, were made on a cryostat (Leica Biosystems) at −20 °C and mounted on pre-chilled slides, which were vapor fixed for 24 h at −20 °C by inversion in pre-chilled 37% formaldehyde in a closed staining dish while keeping sections in the vapor phase. Sections were then post-fixed in the liquid phase, by inverting slides again so sections rested in the 37% formaldehyde for 30 min. Sections were then re-hydrated in PBS until staining. Confocal imaging used Zeiss LSM II Airyscan microscope.

### Fatty acid uptake assay

The fatty acid uptake real-time assay^44^ was performed according to the manufacturer’s protocol (QBT Fatty Acid Uptake Kit, Molecular Devices). The assay was optimized for a 96-well format. Briefly, equal number of hMECs were seeded in black, clear bottom 96-well plates coated with attachment factor (AF S006100, ThermoFisher Scientific). Upon reaching confluence, cells were washed, and serum starved for 4 h. After a gentle wash with PBS the cells were treated either with vehicle control or SSO (100 µM, 30 min), GW4869 (10 µM, 10 min), Latrunculin B (2.5 µM, 30 min), Src inhibitor-1 (10 µM, 2 h), or ROCK inhibitor (10 µM, 30 min). The cells were washed with HBSS, 100 µL HBSS was then added, and basal fluorescence measured with a pre-warmed (37 °C) microplate reader (bottom read, excitation 488/emission 515, filter cutoff 495 nm, 21 s read). With a multichannel pipette, HBSS was removed, 100 µL warmed QBT (protected from light) added, and fluorescence measured. Background reading with HBSS were subtracted from QBT readings to get final fluorescence. Data were plotted as the calculated area under the curve (AUC).

### Immunoprecipitation (IP) and western blotting (WB)

4-h serum-starved MECs were treated with OA:BSA, 100 µM:50 µM (30 min, 37 °C), lysed and processed for IP and or western blotting. Washed cells were scraped into 1 ml IP buffer (0.1 M NaCl, 0.3 M Sucrose, 30 mM MgCl_2_, 10 mM PIPES, 0.5 mM EDTA, 0.1% Nonidet P-40 with protease and phosphatase inhibitors (Roche) and 0.5 mM pervanadate), kept on ice for 20 min then lysed by passage 5 times through a 25-gauge needle. Lysates were clarified (11,000 × *g*, 10 min) and aliquots (50 µg protein) incubated for 5 h with CD36 antibody (R & D Systems) coupled to protein G magnetic beads (Dynabeads). Goat IgG-coupled beads were used as controls. Immune complexes were separated magnetically, IP buffer-washed, and boiled in 50 µL of 2X SDS-Sample buffer prior to SDS-PAGE (4–12% ThermoFisher Scientific).

For WBs, cells were scraped and lysed 20 min in ice-cold RIPA buffer (20 mM Tris-HCl (pH 7.5), 150 mM NaCl, 1 mM Na_2_EDTA, 1 mM EGTA, 1% NP-40, 1% sodium deoxycholate, 2.5 mM sodium pyrophosphate, 1 mM beta-glycerophosphate, 1 mM Na_3_VO_4_, 1 µg/mL leupeptin, 1 mM PMSF, and 1 μg/mL protease inhibitor mix). Cleared lysates (10,000 × *g*, 10 min) were assayed for protein content (DC Protein Assay, Bio-Rad) and proteins (30 µg) separated on 4%–20% gradient gels, transferred to polyvinylidene fluoride membranes (Immobilon Fl, Millipore), blocked (1X TBS, 0.25% fish gelatin, 0.01% Na-azide, 0.05% Tween-20) and incubated overnight at 4 °C with primary antibodies. Washed membranes were incubated 1 h with infrared dye-labeled secondary antibodies (RT, LI-COR Biotechnology) and imaged (LI-COR Odyssey Infrared, Biosciences). Antibodies are listed in Supplementary Table 3.

### Determination of filamentous to globular (F/G) actin ratio

Cell lysates (CL) in ice-cold PHEM buffer (60 mM PIPES, 20 mM HEPES, 10 mM EGTA, 2 mM MgCl_2_ and 1% Triton X-100; pH 7.0) were centrifuged (10 min, 10,000 rpm)^107^. After SDS-PAGE (4%–12% gels) Supernatant (G actin) and pellet (F actin) were probed with β-actin (1:10,000; Santa Cruz) and infrared conjugated antibodies (1:10,000, LI-COR Biosciences). Signal detection used LI-COR Odyssey and Image Studio Lite (LI-COR Biosciences).

### Transmission electron microscopy (TEM)

Subcellular visualization of palmitic acid in endothelial cells used palmitoyl nanogold (Nanoprobes, Yaphank, NY) for 10 min before fixing cells with 4% PFA (Polysciences Inc., Warrington, PA) in 100 mM PIPES/0.5 mM MgCl_2_, pH 7.2 for 1 h at 4 °C. Samples embedded in 10% gelatin were incubated overnight at 4 °C in 2.3 M sucrose/20% polyvinyl pyrrolidone in PIPES/MgCl_2_, then trimmed, frozen in liquid nitrogen and sectioned (Ultracut UCT7 cryo-ultramicrotome Leica Microsystems Inc., Bannockburn, IL). Ultrathin sections (50 nm) placed on nickel grids freshly coated with glow discharged carbon, were washed in PIPES buffer and incubated with GoldEnhance EM Plus (Nanoprobes) per the manufacturer’s instructions. Development times determined 4 min as optimal for gold deposition onto nanogold particles and minimal background. Grids were rinsed extensively in deionized water, stained with 0.3% uranyl acetate/2% methyl cellulose, and viewed on a JEOL 1200 EX transmission electron microscope (JEOL USA Inc., Peabody, MA) with an AMT 8-megapixel digital camera and AMT Image Capture Engine V602 (Advanced Microscopy Techniques, Woburn, MA).

### Isolation and characterization of sEV

EC conditioned media were centrifuged to remove cell debris (2000 × *g*, 20 min) and microvesicles (15,000 × *g*, 30 min). Post-microvesicle supernatant was incubated with ExoQuick TC (Systems Biosciences) overnight at 4 °C and centrifuged (1500 × *g*, 30 min) to pellet sEVs. The pellets were resuspended with PBS for Nanoparticle Tracking Analysis (Nanosight NS300, Malvern Pananalytical) or in 1X RIPA buffer for western blot and marker analysis. The sEVs from cells incubated with nanogold palmitic acid were subjected to TEM as above.

### Isolation and characterization of ^3^H-oleate loaded sEV

mMECs and hMECS incubated with ^3^H-oleate solution complexed with BSA. sEVs were isolated as described above. sEV were dissolved in chloroform/methanol (1:1, by vol) and lipids were separated by thin layer chromatography using petroleum ether/diethyl ether/acetic acid (70:30:1, by vol). Lipid classes were visualized by exposure to iodine vapor, the corresponding bands scraped, and associated radioactivity determined by liquid scintillation counting.

### Uptake of EC-generated FA-sEVs by myotubes in transwells

For visualizing sEV transfer from ECs to myotubes, hMECs were transduced with RFP-CD63 lentiviral particles (Systems Bioscience). After 7 days of antibiotic selection the cells were grown on collagen-coated transwells (Falcon, 0.4 µm pore) in 24-well plates until 3–5 days post confluence, assessed by trans-endothelial electric resistance (TEER)^80^ using EVOM2 (World Precision Instruments). Barrier integrity and impermeability to FAs was also assessed using two fluorescent probes, Dextran-FITC (70 kD, Sigma) and C12 Bodipy (Life Technologies). The post confluent hMECs on transwell filters were serum starved (2 h) before adding Dextran-FITC (1 mg/mL), OA (200 µM OA, 40 µM FA-Free BSA) and/or C12-Bodipy (20 µM, 40 µM FA-Free BSA) to the upper chamber (100 µL mix per well). Regular growth media (600 µL) was placed in the lower chamber and media was sampled for measurement of Dextran-FITC (485/520 ex/em) or C12-Bodipy (530/590 ex/em). To compute Dextran-FITC and C12-Bodipy recovered in the lower chamber as percent of input to the upper chamber, a standard curve was prepared by linear regression of serial dilutions of Dextran-FITC and C12-Bodipy input solutions.

Human skeletal muscle myoblasts (hSMM) on coverslips in skeletal muscle growth media (Lonza) were grown and differentiated into myotubes^55^ below the transwells. Serum-starved (4 h) hMECs were treated with alkyne OA (25 µM) added to upper chamber and bottom myotubes were collected 15 or 30 min later for immunofluorescence (Zeiss LSM 880 Airyscan Confocal Microscope, 40X or 63X). Images were processed using Zeiss Zen (Blue) Imaging. ImageJ was used to determine mean intensity.

### Myotube treatment with hMEC-generated FA-sEVs

hMECs grown to confluence were serum-starved and treated with OA:BSA (100 µM:50 µM) or BSA (50 µM controls) and the sEVs collected from media over 48 h. Particle number and protein content were determined before addition to myotubes. Human myoblasts (hSMM) in 6-well plates were differentiated into myotubes^55^, serum-starved and treated with 5 × 10^8^ sEVs/well for 2 h. Myotube RNA (RNeasy mini kit, Qiagen) was reverse-transcribed (SuperScript VILO Mastermix, ThermoFisher Scientific), quantified (RT-PCR, power SYBR and QuantStudio 3, Applied Biosystems, ThermoFisher Scientific) and levels normalized to housekeeping gene 36B4. Primers used are in Supplementary Table 4.

### RNASeq on control and CD36-knockdown hMEC

hMECs were treated with either control or CD36 siRNA as above. 72 h after treatment, total RNA was isolated using TRIzol Reagent (ThermoFisher Scientific) followed by column-based sample cleanup (RNeasy, Qiagen). Samples were submitted to the Washington University Genome Technology Access Center (GTAC) for library preparation, sequencing and read alignment. Normalization and differential gene expression was determined using the integrated Differential Expression and Pathway Analysis (iDEP) web platform^108^.

### Preparation and LC/MS analysis of sEV lipids

Samples were collected and aliquots representing the same EV counts were dried under nitrogen and resuspended in 2:2:1 acetonitrile:methanol:H_2_O (v/v) (for polar metabolites, e.g., fatty acids, phospholipids) or in 100% isopropanol (for less-polar metabolites, e.g., ceramides). The samples were mixed on an orbital shaker (360 rpm) for 1 min at room temperature before the LC/MS analysis.

LC/MS analysis was performed on an Agilent 1290 Infinity II LC-system coupled to an Agilent 6545 QTOF mass spectrometer with a dual Agilent Jet Stream electrospray ionization source. Analysis of ceramides and fatty acids was further verified in high resolution using an Orbitrap ID-X Tribrid mass spectrometer (Thermo Scientific). A Vanquish Horizon UHPLC system, was interfaced with the mass spectrometer via electrospray ionization in both positive and negative ion mode with a spray voltage of 3.5 and 2.8 kV, respectively. Lipids were separated on a CORTECS UPLC C18 column (2.1 × 100 mm, 1.6 µm; part No. 186007095) including a Waters UPLC HSS VanGuard Pre-Column (2.1 × 5 mm, 1.8 µm; part No. 186007949) at a temperature of 60 °C and a flow rate of 250 mL/min. The mobile phases consisted of A: 60% acetonitrile, 40% water, 0.1% formic acid, 10 mM ammonium formate, 2.5 mM medronic acid, and B: 90% 2-propanol, 10% acetonitrile, 0.1% formic acid, 10 mM ammonium formate (in 1 mL water). The following linear gradient was used: 0–2 min, 30% B; 17 min, 75% B; 20 min, 85% B; 23–26 min, 100% B; 26 min, 30% B followed by a re-equilibration phase of 5 min. Lipids were detected in positive ion mode with following source parameters: gas temperature 250 °C, drying gas flow 11 L/min, nebulizer pressure 35 psi, sheath gas temperature 300 °C, sheath gas flow 12 L/min, VCap 3000 V, nozzle voltage 500 V, Fragmentor 160 V, Skimmer 65 V, Oct 1 RF Vpp 750 V, and *m*/*z* range 50–1700. Data were acquired under continuous reference mass correction at *m*/*z* 121.0509 and 922.0890 in positive ion mode. Samples were randomized before analysis and a quality-control (QC) sample was injected to monitor instrument signal stability.

### Acquiring MS/MS data, data processing, and normalization

MS/MS spectra for lipids were acquired using an iterative approach in the MassHunter Acquisition Software (Version 10.1.48, Agilent Technologies) on an Agilent 6545 QTOF. Source settings for MS1 data acquisition were used. MS/MS spectra were acquired at a scan rate of 3 spectra/s with different intensity thresholds and collision energies of 10, 20, and 40 V to increase identification rates. For the ID-X Orbitrap, data were acquired in data-dependent acquisition (DDA) by using the built-in deep scan option (AcquireX) with a mass range of 67–900 *m*/*z*. The MS/MS scans were acquired at 15 K resolution and cross referenced to a library generated from NIST SRM 1950 plasma sample in both positive and negative ion mode with different collision energies in the range of 20 NCE to 50 NCE for HCD and 30 NCE for CID to maximize identifications.

Lipid iterative MS/MS data were annotated with the Agilent Lipid Annotator software. All data files were then analyzed in Skyline-daily (Version 22.2.1.256) to obtain peak areas, *m*/*z* values of the metabolite and lipid target lists, obtained from the metabolite identification workflow, which had at least an MS/MS match to an online library, were extracted under consideration of retention times.

### Statistical analyses

All data presented are representative of at least three independent experiments, unless indicated otherwise. Quantifications are shown as means ± standard errors (S.E.). Statistical significance, *p* value was evaluated using unpaired Student’s *t* test when comparing between two groups. For comparison between more than two groups one-way ANOVA or two-way ANOVA analysis was used followed by post hoc Tukey for multiple comparisons as indicated (****P ≤ 0.0001, ***P ≤ 0.001, **P ≤ 0.01, *P ≤ 0.05, ns *P* > 0.05).

### Reporting summary

Further information on research design is available in the Nature Portfolio Reporting Summary linked to this article.

## Supplementary information

Supplementary Information

Reporting Summary

## Data Availability

Source data files, including uncropped blots, are provided with this paper (Source data file). Lipidomic data generated in this study is deposited at metabolomics workbench and can be accessed at 10.21228/M8PH89. RNA-seq data in this study have been deposited in the Gene Expression Omnibus database under accession code GSE235988. All other relevant data could be obtained from the corresponding authors Vivek Peche and Nada Abumrad upon request. Source data are provided with this paper.

## Source data

Source Data

## References

[1] Ghosh-Choudhary S, Liu J, Finkel T (2020). Metabolic regulation of cell fate and function. Trends Cell Biol..

[2] Hubert M (2020). Lipid accumulation controls the balance between surface connection and scission of caveolae. Elife.

[3] Jacome-Sosa M (2021). CD36 maintains the gastric mucosa and associates with gastric disease. Commun. Biol..

[4] Stern JH, Rutkowski JM, Scherer PE (2016). Adiponectin, leptin, and fatty acids in the maintenance of metabolic homeostasis through adipose tissue crosstalk. Cell Metab..

[5] Goodpaster BH, Sparks LM (2017). Metabolic flexibility in health and disease. Cell Metab..

[6] Abumrad NA, Goldberg IJ (2016). CD36 actions in the heart: lipids, calcium, inflammation, repair and more?. Biochim. Biophys. Acta.

[7] Jang C (2016). A branched-chain amino acid metabolite drives vascular fatty acid transport and causes insulin resistance. Nat. Med..

[8] Bae H (2020). Angiopoietin-2-integrin alpha5beta1 signaling enhances vascular fatty acid transport and prevents ectopic lipid-induced insulin resistance. Nat. Commun..

[9] Son NH (2018). Endothelial cell CD36 optimizes tissue fatty acid uptake. J. Clin. Invest.

[10] Richieri GV, Kleinfeld AM (1995). Unbound free fatty acid levels in human serum. J. Lipid Res..

[11] Goldberg IJ (2021). Lipolytic enzymes and free fatty acids at the endothelial interface. Atherosclerosis.

[12] Mallick R, Basak S, Duttaroy AK (2021). Fatty acids and evolving roles of their proteins in neurological, cardiovascular disorders and cancers. Prog. Lipid Res..

[13] Abumrad NA (2021). Endothelial cell receptors in tissue lipid uptake and metabolism. Circ. Res..

[14] Harmon CM, Abumrad NA (1993). Binding of sulfosuccinimidyl fatty acids to adipocyte membrane proteins: isolation and amino-terminal sequence of an 88-kD protein implicated in transport of long-chain fatty acids. J. Membr. Biol..

[15] Hajri T, Abumrad NA (2002). Fatty acid transport across membranes: relevance to nutrition and metabolic pathology. Annu. Rev. Nutr..

[16] Hames KC, Vella A, Kemp BJ, Jensen MD (2014). Free fatty acid uptake in humans with CD36 deficiency. Diabetes.

[17] Bartelt A (2011). Brown adipose tissue activity controls triglyceride clearance. Nat. Med..

[18] Bharadwaj KG (2010). Chylomicron- and VLDL-derived lipids enter the heart through different pathways: in vivo evidence for receptor- and non-receptor-mediated fatty acid uptake. J. Biol. Chem..

[19] He C (2018). NanoSIMS analysis of intravascular lipolysis and lipid movement across capillaries and into cardiomyocytes. Cell Metab..

[20] Canton J, Neculai D, Grinstein S (2013). Scavenger receptors in homeostasis and immunity. Nat. Rev. Immunol..

[21] Hsieh FL (2016). The structural basis for CD36 binding by the malaria parasite. Nat. Commun..

[22] Kuda O (2013). Sulfo-N-succinimidyl oleate (SSO) inhibits fatty acid uptake and signaling for intracellular calcium via binding CD36 lysine 164: SSO also inhibits oxidized low density lipoprotein uptake by macrophages. J. Biol. Chem..

[23] Pepino MY, Kuda O, Samovski D, Abumrad NA (2014). Structure-function of CD36 and importance of fatty acid signal transduction in fat metabolism. Annu. Rev. Nutr..

[24] Coppiello G (2015). Meox2/Tcf15 heterodimers program the heart capillary endothelium for cardiac fatty acid uptake. Circulation.

[25] Kalluri AS (2019). Single-cell analysis of the normal mouse aorta reveals functionally distinct endothelial cell populations. Circulation.

[26] Kanda T (2009). PPARgamma in the endothelium regulates metabolic responses to high-fat diet in mice. J. Clin. Invest.

[27] Coburn CT (2000). Defective uptake and utilization of long chain fatty acids in muscle and adipose tissues of CD36 knockout mice. J. Biol. Chem..

[28] Nolan DJ (2013). Molecular signatures of tissue-specific microvascular endothelial cell heterogeneity in organ maintenance and regeneration. Dev. Cell.

[29] Conchinha NV (2021). Protocols for endothelial cell isolation from mouse tissues: brain, choroid, lung, and muscle. STAR Protoc..

[30] Ren B, Hale J, Srikanthan S, Silverstein RL (2011). Lysophosphatidic acid suppresses endothelial cell CD36 expression and promotes angiogenesis via a PKD-1-dependent signaling pathway. Blood.

[31] Huang J, Stohl LL, Zhou X, Ding W, Granstein RD (2011). Calcitonin gene-related peptide inhibits chemokine production by human dermal microvascular endothelial cells. Brain Behav. Immun..

[32] Orecchia A (2011). Sirtinol treatment reduces inflammation in human dermal microvascular endothelial cells. PLoS ONE.

[33] Thiele C, Wunderling K, Leyendecker P (2019). Multiplexed and single cell tracing of lipid metabolism. Nat. Methods.

[34] Wunderling K (2021). Hepatic synthesis of triacylglycerols containing medium-chain fatty acids is dominated by diacylglycerol acyltransferase 1 and efficiently inhibited by etomoxir. Mol. Metab..

[35] Thiele C (2012). Tracing fatty acid metabolism by click chemistry. ACS Chem. Biol..

[36] Laval T (2021). De novo synthesized polyunsaturated fatty acids operate as both host immunomodulators and nutrients for Mycobacterium tuberculosis. Elife.

[37] Gallion LA (2022). “Fix and Click” for assay of sphingolipid signaling in single primary human intestinal epithelial cells. Anal. Chem..

[38] Abumrad NA (2001). Assay of membrane transport of long-chain fatty acids by adipocytes. Methods Mol. Biol..

[39] Dunn KW, Kamocka MM, McDonald JH (2011). A practical guide to evaluating colocalization in biological microscopy. Am. J. Physiol. Cell Physiol..

[40] Trigatti BL, Anderson RG, Gerber GE (1999). Identification of caveolin-1 as a fatty acid binding protein. Biochem. Biophys. Res. Commun..

[41] Frank PG, Pavlides S, Lisanti MP (2009). Caveolae and transcytosis in endothelial cells: role in atherosclerosis. Cell Tissue Res..

[42] Liu P, Rudick M, Anderson RG (2002). Multiple functions of caveolin-1. J. Biol. Chem..

[43] Coort SL (2002). Sulfo-N-succinimidyl esters of long chain fatty acids specifically inhibit fatty acid translocase (FAT/CD36)-mediated cellular fatty acid uptake. Mol. Cell Biochem..

[44] Liao J, Sportsman R, Harris J, Stahl A (2005). Real-time quantification of fatty acid uptake using a novel fluorescence assay. J. Lipid Res..

[45] Li H, Black PN, DiRusso CC (2005). A live-cell high-throughput screening assay for identification of fatty acid uptake inhibitors. Anal. Biochem..

[46] Fridolfsson HN, Roth DM, Insel PA, Patel HH (2014). Regulation of intracellular signaling and function by caveolin. FASEB J..

[47] Shvets E, Bitsikas V, Howard G, Hansen CG, Nichols BJ (2015). Dynamic caveolae exclude bulk membrane proteins and are required for sorting of excess glycosphingolipids. Nat. Commun..

[48] Frank PG, Woodman SE, Park DS, Lisanti MP (2003). Caveolin, caveolae, and endothelial cell function. Arterioscler Thromb. Vasc. Biol..

[49] Zhang X, Fernandez-Hernando C (2020). Transport of LDLs into the arterial wall: impact in atherosclerosis. Curr. Opin. Lipido..

[50] Hao JW (2020). CD36 facilitates fatty acid uptake by dynamic palmitoylation-regulated endocytosis. Nat. Commun..

[51] Ring A, Le Lay S, Pohl J, Verkade P, Stremmel W (2006). Caveolin-1 is required for fatty acid translocase (FAT/CD36) localization and function at the plasma membrane of mouse embryonic fibroblasts. Biochim. Biophys. Acta.

[52] del Pozo MA (2005). Phospho-caveolin-1 mediates integrin-regulated membrane domain internalization. Nat. Cell Biol..

[53] Zimnicka AM (2016). Src-dependent phosphorylation of caveolin-1 Tyr-14 promotes swelling and release of caveolae. Mol. Biol. Cell.

[54] Bull HA, Brickell PM, Dowd PM (1994). Src-related protein tyrosine kinases are physically associated with the surface antigen CD36 in human dermal microvascular endothelial cells. FEBS Lett..

[55] Samovski D (2018). Regulation of insulin receptor pathway and glucose metabolism by CD36 signaling. Diabetes.

[56] Silverstein RL, Li W, Park YM, Rahaman SO (2010). Mechanisms of cell signaling by the scavenger receptor CD36: implications in atherosclerosis and thrombosis. Trans. Am. Clin. Climatol. Assoc..

[57] Joshi B (2012). Phosphocaveolin-1 is a mechanotransducer that induces caveola biogenesis via Egr1 transcriptional regulation. J. Cell Biol..

[58] Thery C (2018). Minimal information for studies of extracellular vesicles 2018 (MISEV2018): a position statement of the International Society for Extracellular Vesicles and update of the MISEV2014 guidelines. J. Extracell. Vesicles.

[59] Trajkovic K (2008). Ceramide triggers budding of exosome vesicles into multivesicular endosomes. Science.

[60] Canals D (2010). Differential effects of ceramide and sphingosine 1-phosphate on ERM phosphorylation: probing sphingolipid signaling at the outer plasma membrane. J. Biol. Chem..

[61] Parton RG, Kozlov MM, Ariotti N (2020). Caveolae and lipid sorting: Shaping the cellular response to stress. J. Cell Biol..

[62] Elsherbini A, Bieberich E (2018). Ceramide and exosomes: a novel target in cancer biology and therapy. Adv. Cancer Res..

[63] Milhas D, Clarke CJ, Hannun YA (2010). Sphingomyelin metabolism at the plasma membrane: implications for bioactive sphingolipids. FEBS Lett..

[64] Airola, M. V. & Hannun, Y. A. Sphingolipid metabolism and neutral sphingomyelinases. *Handb. Exp. Pharmacol*. **215**, 57–76 (2013). 10.1007/978-3-7091-1368-4_3 PMC4043343 23579449

[65] Kosaka N (2013). Neutral sphingomyelinase 2 (nSMase2)-dependent exosomal transfer of angiogenic microRNAs regulate cancer cell metastasis. J. Biol. Chem..

[66] Ratitong B, Marshall M, Pearlman E (2021). beta-Glucan-stimulated neutrophil secretion of IL-1alpha is independent of GSDMD and mediated through extracellular vesicles. Cell Rep..

[67] Luberto C (2002). Inhibition of tumor necrosis factor-induced cell death in MCF7 by a novel inhibitor of neutral sphingomyelinase. J. Biol. Chem..

[68] Airola MV (2017). Structure of human nSMase2 reveals an interdomain allosteric activation mechanism for ceramide generation. Proc. Natl Acad. Sci. USA.

[69] Choezom D, Gross JC (2022). Neutral sphingomyelinase 2 controls exosome secretion by counteracting V-ATPase-mediated endosome acidification. J. Cell Sci..

[70] Crewe C (2018). An endothelial-to-adipocyte extracellular vesicle axis governed by metabolic state. Cell.

[71] Guo BB, Bellingham SA, Hill AF (2015). The neutral sphingomyelinase pathway regulates packaging of the prion protein into exosomes. J. Biol. Chem..

[72] Li J (2013). Exosomes mediate the cell-to-cell transmission of IFN-alpha-induced antiviral activity. Nat. Immunol..

[73] Sharma A (2015). Direct endothelial nitric oxide synthase activation provides atheroprotection in diabetes-accelerated atherosclerosis. Diabetes.

[74] Tojkander S, Gateva G, Lappalainen P (2012). Actin stress fibers-assembly, dynamics and biological roles. J. Cell Sci..

[75] Rustom A, Saffrich R, Markovic I, Walther P, Gerdes HH (2004). Nanotubular highways for intercellular organelle transport. Science.

[76] Haimovich G (2017). Intercellular mRNA trafficking via membrane nanotube-like extensions in mammalian cells. Proc. Natl Acad. Sci. USA.

[77] Fujiwara I, Zweifel ME, Courtemanche N, Pollard TD (2018). Latrunculin A accelerates actin filament depolymerization in addition to sequestering actin monomers. Curr. Biol..

[78] Peche V (2007). CAP2, cyclase-associated protein 2, is a dual compartment protein. Cell Mol. Life Sci..

[79] Hartmann S, Ridley AJ, Lutz S (2015). The function of Rho-associated kinases ROCK1 and ROCK2 in the pathogenesis of cardiovascular disease. Front. Pharm..

[80] Srinivasan B (2015). TEER measurement techniques for in vitro barrier model systems. J. Lab Autom..

[81] Stahl PD, Raposo G (2018). Exosomes and extracellular vesicles: the path forward. Essays Biochem..

[82] Kilinc S (2021). Oncogene-regulated release of extracellular vesicles. Dev. Cell.

[83] Agbu P, Carthew RW (2021). MicroRNA-mediated regulation of glucose and lipid metabolism. Nat. Rev. Mol. Cell Biol..

[84] McCann JV (2020). Reporter mice for isolating and auditing cell type-specific extracellular vesicles in vivo. Genesis.

[85] Harris LA (2015). Perilipin 5-driven lipid droplet accumulation in skeletal muscle stimulates the expression of fibroblast growth factor 21. Diabetes.

[86] Hajri T, Han XX, Bonen A, Abumrad NA (2002). Defective fatty acid uptake modulates insulin responsiveness and metabolic responses to diet in CD36-null mice. J. Clin. Invest.

[87] Glatz JFC, Nabben M, Luiken J (2022). CD36 (SR-B2) as master regulator of cellular fatty acid homeostasis. Curr. Opin. Lipido..

[88] Jones JH, Minshall RD (2022). Endothelial transcytosis in acute lung injury: emerging mechanisms and therapeutic approaches. Front. Physiol..

[89] Tait JF, Smith C (1999). Phosphatidylserine receptors: role of CD36 in binding of anionic phospholipid vesicles to monocytic cells. J. Biol. Chem..

[90] Garcia NA (2019). Circulating exosomes deliver free fatty acids from the bloodstream to cardiac cells: Possible role of CD36. PLoS ONE.

[91] Glatz JFC, Wang F, Nabben M, Luiken J (2021). CD36 as a target for metabolic modulation therapy in cardiac disease. Expert Opin. Ther. Targets.

[92] Schulze PC, Drosatos K, Goldberg IJ (2016). Lipid use and misuse by the heart. Circ. Res..

[93] Fritzen AM, Lundsgaard AM, Kiens B (2020). Tuning fatty acid oxidation in skeletal muscle with dietary fat and exercise. Nat. Rev. Endocrinol..

[94] Fiorenza M, Gliemann L, Brandt N, Bangsbo J (2020). Hormetic modulation of angiogenic factors by exercise-induced mechanical and metabolic stress in human skeletal muscle. Am. J. Physiol. Heart Circ. Physiol..

[95] van Niel G, D’Angelo G, Raposo G (2018). Shedding light on the cell biology of extracellular vesicles. Nat. Rev. Mol. Cell Biol..

[96] Grevengoed TJ, Klett EL, Coleman RA (2014). Acyl-CoA metabolism and partitioning. Annu. Rev. Nutr..

[97] Storch J, Thumser AE (2000). The fatty acid transport function of fatty acid-binding proteins. Biochim. Biophys. Acta.

[98] Glatz JFC, Luiken J (2018). Dynamic role of the transmembrane glycoprotein CD36 (SR-B2) in cellular fatty acid uptake and utilization. J. Lipid Res..

[99] Glatz JFC, Luiken J, Nabben M (2020). CD36 (SR-B2) as a target to treat lipid overload-induced cardiac dysfunction. J. Lipid Atheroscler..

[100] Guo H (2017). Atg5 disassociates the V1V0-ATPase to promote exosome production and tumor metastasis independent of canonical macroautophagy. Dev. Cell.

[101] Xu S (2021). Uptake of oxidized lipids by the scavenger receptor CD36 promotes lipid peroxidation and dysfunction in CD8(+) T cells in tumors. Immunity.

[102] Chaurasia B, Summers SA (2021). Ceramides in metabolism: key lipotoxic players. Annu. Rev. Physiol..

[103] Febbraio M (1999). A null mutation in murine CD36 reveals an important role in fatty acid and lipoprotein metabolism. J. Biol. Chem..

[104] Sorensen I, Adams RH, Gossler A (2009). DLL1-mediated Notch activation regulates endothelial identity in mouse fetal arteries. Blood.

[105] Essandoh K (2015). Blockade of exosome generation with GW4869 dampens the sepsis-induced inflammation and cardiac dysfunction. Biochim. Biophys. Acta.

[106] Taniguchi K (2013). Inhibition of Src kinase blocks high glucose-induced EGFR transactivation and collagen synthesis in mesangial cells and prevents diabetic nephropathy in mice. Diabetes.

[107] Rust MB (2010). Learning, AMPA receptor mobility and synaptic plasticity depend on n-cofilin-mediated actin dynamics. EMBO J..

[108] Ge SX, Son EW, Yao R (2018). iDEP: an integrated web application for differential expression and pathway analysis of RNA-Seq data. BMC Bioinforma..

[109] Berglund ED (2008). Glucose metabolism in vivo in four commonly used inbred mouse strains. Diabetes.

[110] Goren HJ, Kulkarni RN, Kahn CR (2004). Glucose homeostasis and tissue transcript content of insulin signaling intermediates in four inbred strains of mice: C57BL/6, C57BLKS/6, DBA/2, and 129X1. Endocrinology.

