# Supplementary material for: Endothelial cell CD36 regulates membrane ceramide formation, exosome fatty acid transfer and circulating fatty acid levels
Source: Nat Commun. 2023 Jul 7;14:4029. doi: 10.1038/s41467-023-39752-3 (PMC10329018; doi:10.1038/s41467-023-39752-3)
Supplement: Supplementary file 1 — Supplementary Information [file 41467_2023_39752_MOESM1_ESM.pdf]

**Supplemental Information.**

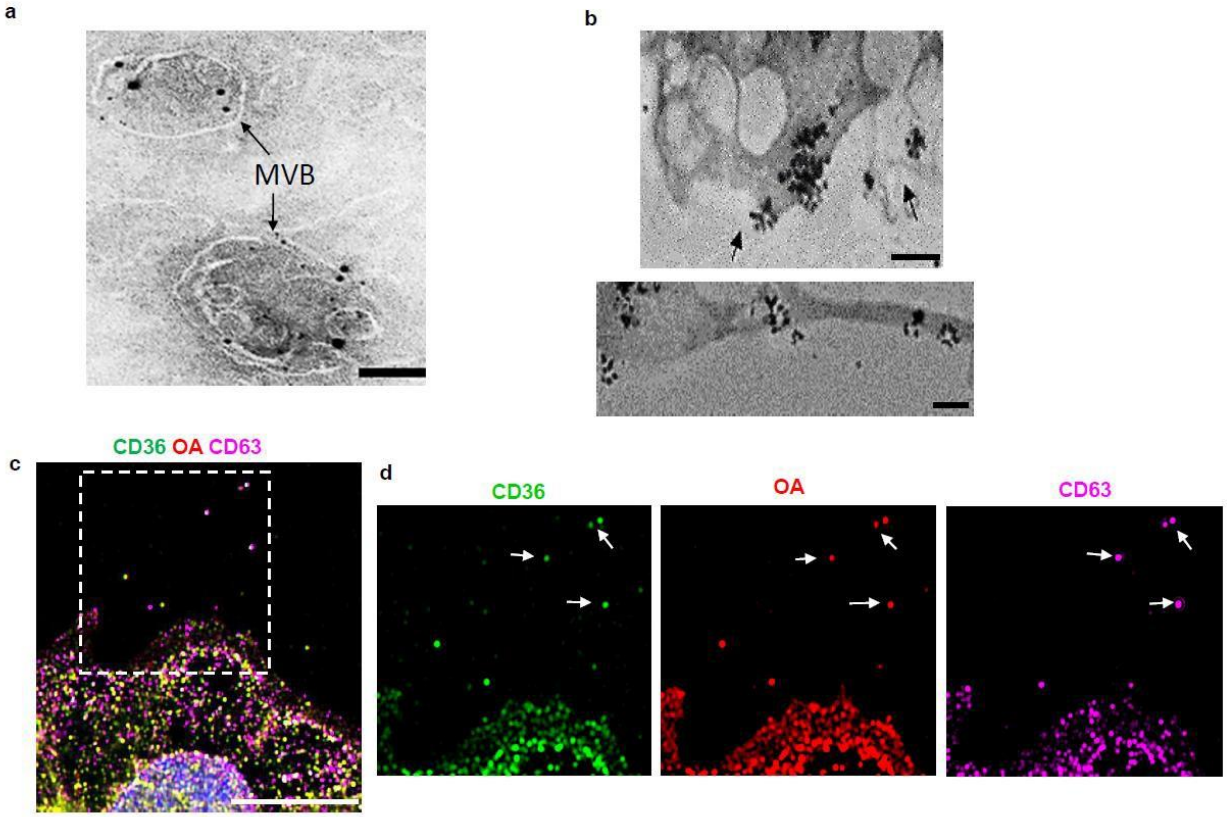


**Supplement Figure 1. Electron microscopy of nanogold palmitic acid in hMECs**

**a.** Nanogold PA, 10µM, 15min, added to hMECs is identified in multi-vesicular bodies (MVBs) inside the cells. Scale bar: 100nm. **b.** EM image showing nanogold PA vesicles being released at the basolateral membrane. Scale bar: 100nm. **c-d.** hMECs treated with alkyne OA, clicked with azide 555, stained for CD36 and CD63, and imaged by confocal microscopy. White arrows: sEVs positive for CD36, OA and CD63. Scale Bar: 10um.

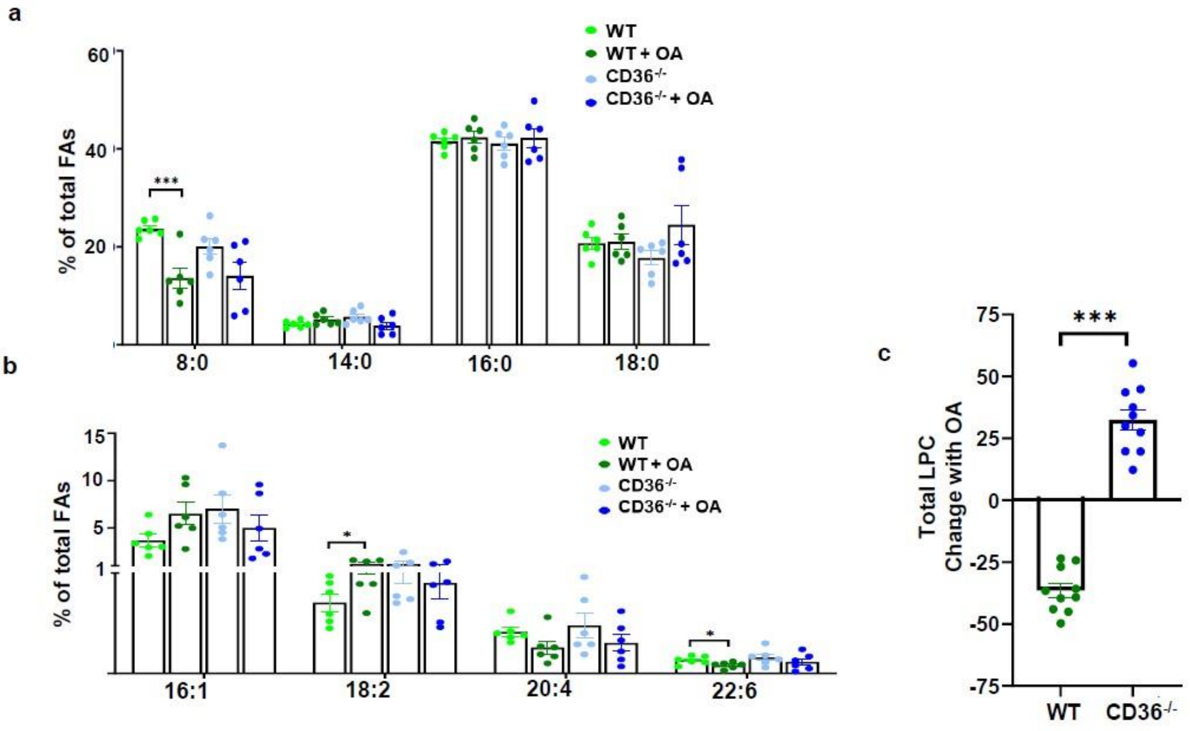


**Supplement Fig. 2. Lipidomic analyses of sEV secreted by mMEC isolated from wildtype and *Cd36$^{-/-}$* mice with and without OA treatment**.

**a-b.** Oleic acid treatment increases levels of free linoleic acid (FA, 18:2, n=6 independent preparations, *p<0.05 by one way ANOVA adjusted for multiple comparison, Data are are means +/- SEM) and decreases levels of free docosahexanoic acid (FA, 22:6, n=6 independent preparations, *p<0.05,one way ANOVA adjusted for multiple comparison, Data are presented as mean values +/- SEM) and caprylic acid (FA, 8:0, n=6 independent preparations, ***p<0.001,one way ANOVA adjusted for multiple comparison, Data are presented as mean values +/- SEM) in sEVs from WT mMECs. n=6 preparations. **c.** Total lysophosphatidylcholine levels (LPC) increased in mMEC sEVs from *Cd36$^{-/-}$* mMECs compared to wildtype mMECs. n= 10 independent preparations. *p <0.05, ***p<0.001 ny unpaired t test. Data are presented as mean values +/- SEM.

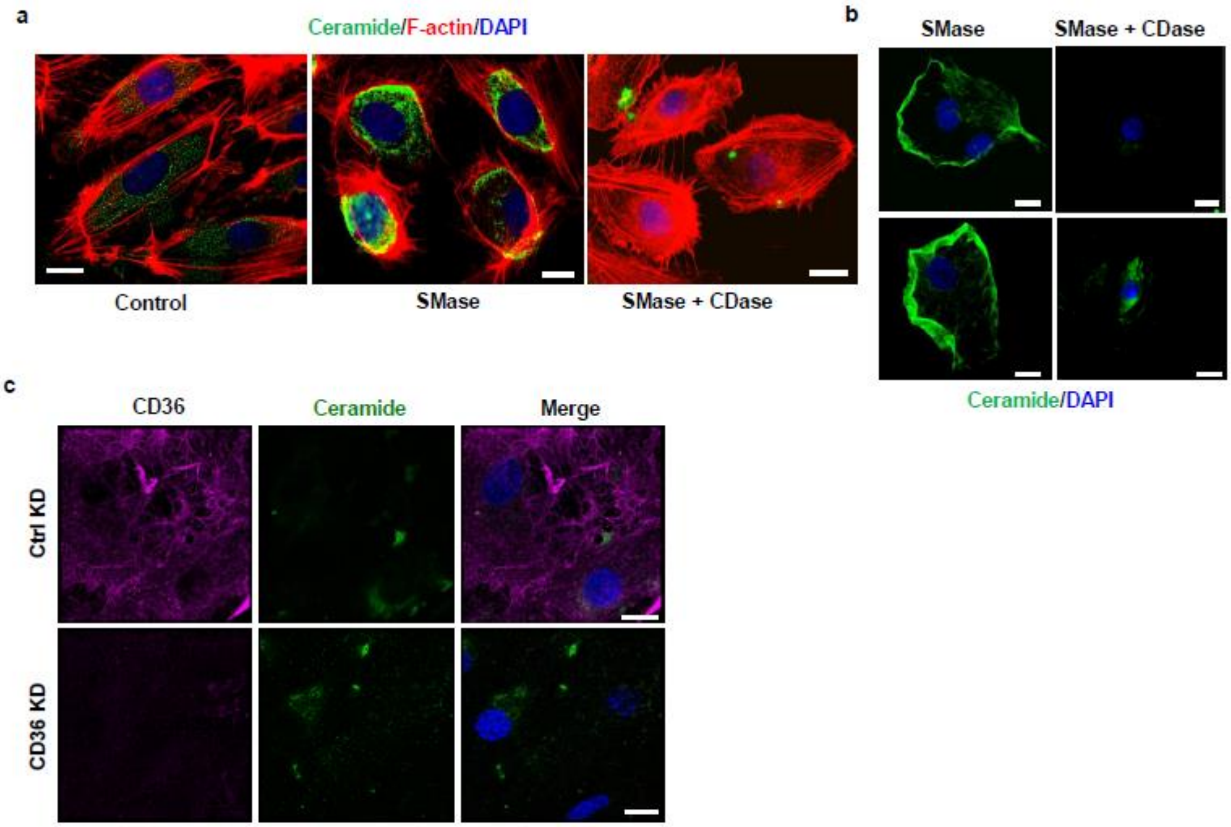


**Supplement Fig. 3. Specificity of ceramide immunostaining**.

**a-b.** hMEC were treated with bacterial Smase (25milliunits,10min) without or with subsequent treatment by ceramidase (CDase, 20milliunits,10min). Cells were fixed, permeabilized and processed for immunofluorescence. Mouse monoclonal ceramide antibody was used followed by goat anti-mouse antibody conjugated to Alexa Fluor 488 (green). F-actin and nuclei visualized with TRITC-Phalloidin and DAPI, respectively. Scale bar: 10µm. **c.** Low basal levels of ceramide in unstimulated hMECs (-OA alkyne). Nuclei were visualized with DAPI. Scale bar: 10µm. Representative of more than 3 preparations.

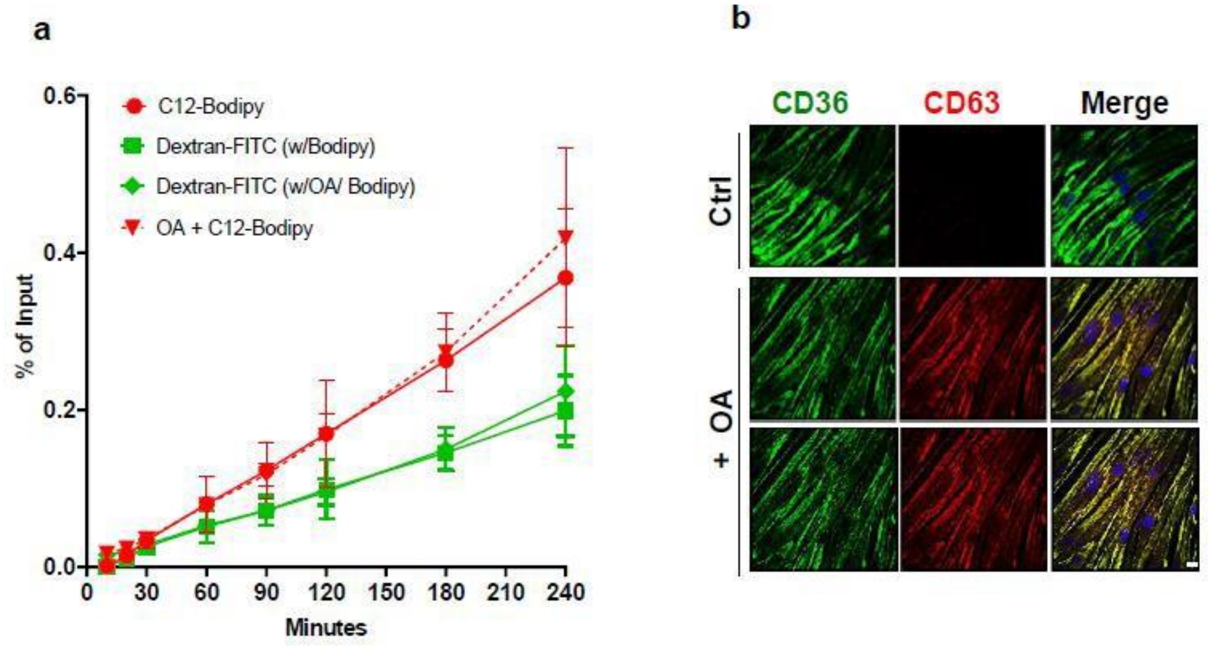


**Supplement Fig. 4. Barrier integrity of hMECs grown on transwells**.

**a.** FITC dextran (1mg/ml) or C12 Bodipy (20uM) were added to the upper chamber and fluorescence for both monitored in the lower chamber. Transfer of dextran or C12 Bodipy was calculated as percent of input to the upper chamber. Dextran transfer was low ~0.02% of input at 30 min and remained <0.2% at 240min. The % dextran transferred was unaffected by addition of oleic acid, OA: albumin, 200:40µM. n=3 independent experiments. Data are means +/- SEM. **b.** *OA induces transfer of the exosome marker RFP-CD63 from hMEC to myotubes*. The hMECs transduced with RFP-CD63 lentivirus were grown on transwell filters until 3 days post confluence. Then human myoblasts grown and differentiated into myotubes on coverslips were placed in the bottom wells. OA was added, OA-BSA:100µM:50µM (30min) then myotubes processed for CD36 immunostaining and confocal microscopy. Scale bar: 10µm.

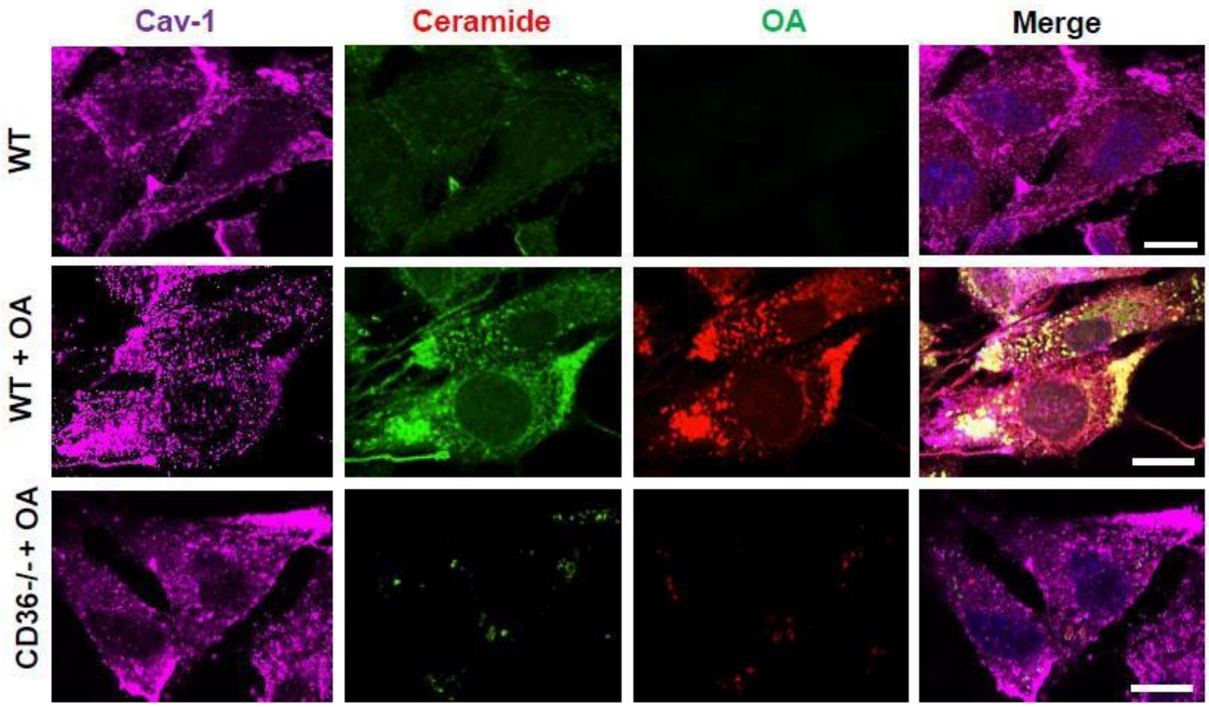


**Supplement Fig. 5**. **Oleic acid addition induces ceramide formation in primary mouse lung microvascular cells**.

mMECs isolated from lungs of WT and *Cd36*$^{-/-}$ mice were treated 10min with 15µM alkyne OA, fixed, clicked for visualizing OA then immunostained for Cav-1 and ceramide. Upper: mMEC controls, middle: OA-treated mMECs. lower: OA-treated *Cd36*$^{-/-}$ mMECs. n= 3 preparations per genotype. Scale bar: 10µm

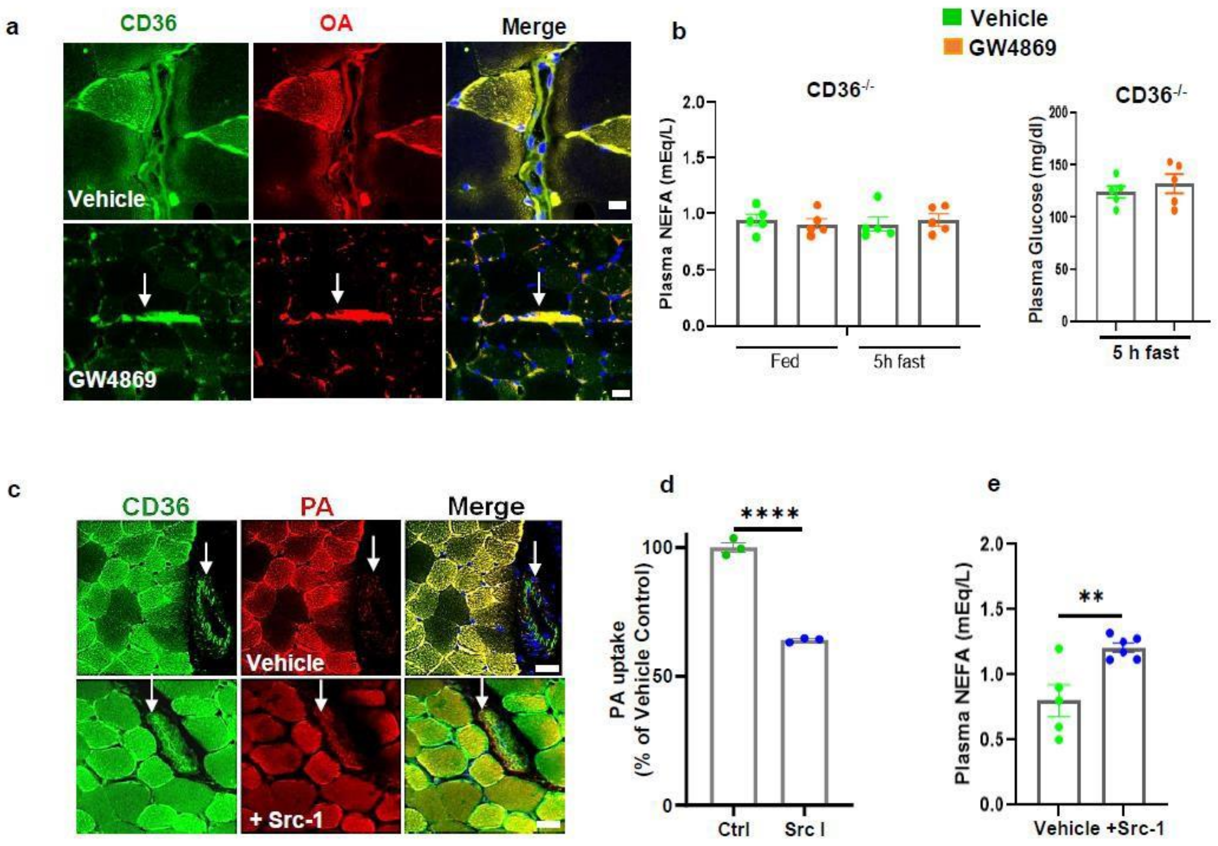


**Supplement Fig. 6**. **Endothelial cells transfer FA to muscle via sEVs.**

**a**. *Blocking sEV secretion by GW4899 reduces muscle FA uptake* in WT mice. The mice (n=3/group) were given intraperitoneally vehicle or GW4869 (2.5µg/g) prior to retro-orbital injection of alkyne OA, and euthanized 30min later. Skeletal muscles were fixed, clicked for OA and stained for CD36. Scale bar 20µm. **b**. *GW4869 does not affect blood nonesterified FAs (NEFAs) or glucose in Cd36$^{-/-}$ mice*. Mice were tested, either fed (end of dark period) or after a 5h fast. NEFAs or glucose were measured in tail vein blood. NEFA and plasma glucose: n=5/group, representative of 2 cohorts. p=ns by two-way Anova adjusted for multiple comparison for NEFA, and unpaired t test for glucose. Data are means +/- SEM. **c**. *Src inhibitor 1 reduces PA uptake by muscle fibers and associates*

*with more OA remaining in blood vessels.* Wildtype mice were given vehicle (DMSO) or Src inhibitor-1 (2mg/kg intraperitoneally for 4 days) then were retro-orbitally injected with alkyne PA and euthanized 30min later. Skeletal muscle sections were processed for click reaction and immunostaining. Upper: Skeletal muscle from a vehicle treated mouse shows strong alkyne-PA uptake into fibers with no detectable PA in a nearby CD36-expressing vessel (arrows). Lower: Skeletal muscle from a Src-1 treated mouse showing reduced PA uptake into fibers and PA retention within a nearby CD36-expressing vessel (arrows). Scale bar 20µm. **d**. PA uptake quantified for data in c. n= 3 mice per condition, $p<0.0001$ by unpaired t test. **e.** *Src inhibition increases blood nonesterified FA (NEFA).* Plasma NEFA were measured in tail blood. n=5-6 mice/group, **$p<0.01$ by unpaired t test. Data are means +/- SEM.

**Supplementary Table 1:** Changes in expression of genes related to endocytosis, vesicular traffic, and cell structure remodeling in human microvascular endothelial cells with CD36 knockdown.

| Gene Name | Gene details | KD/Ctrl fold change | p value |
|---|---|---|---|
| FSCN3 | Fascin, actin-bundling protein 3 | 17.105 | 0.0000903 |
| ITGAD | Integrin subunit alpha D | 7.115 | 0.034874 |
| ADCY8 | Adenylate cyclase 8 | 5.447 | 0.042693 |
| ITGA4 | Integrin subunit alpha 4 | 3.411 | 0.006861 |
| ADCY7 | Adenylate cyclase 7 | 3.092 | 0.015564 |
| CLDN14 | Claudin 14, cell migration | 2.253 | 0.021665 |
| RAB36 | RAB36, vesicular traffic | 2.145 | 0.0000402 |
| ADCY1 | Adenylate cyclase 1 | 1.946 | 0.0263 |
| PI3KCD | PI-4,5-bisphosphate 3-kinase catalytic delta | 1.715 | 0.00072 |
| PTP4A3 | Protein tyrosine phosphatase type IVA, member 3 | 1.691 | 0.007661 |
| ITGAV | Integrin subunit alpha V | 1.668 | 0.000216 |
| ITGB5 | Integrin subunit beta 5 | 1.654 | 0.000818 |
| EHD3 | EH domain containing 3, endocytosis | 1.564 | 0.004321 |
| MLCK | Myosin light chain kinase, actin fibers organization | 1.535 | 0.003951 |
| VPS37B | VPS37B, ESCRT-I subunit, cargo sorting into MVBs | 1.48 | 0.01177 |
| PFN2 | Profilin 2, actin polymerization | 1.446 | 0.000669 |
| DNM3 | Dynamin 3, endocytosis, vesicular traffic | 1.415 | 0.037675 |
| CLDN12 | Claudin 12, cell migration | 1.395 | 0.00106 |
| CORO7 | Coronin 7, Actin binding, Golgi to endosome transport | 1.368 | 0.014585 |
| TFAM | Transcription factor A, mitochondrial, late endosomes/lysosomes | 0.433 | 0.0000408 |
| RAB3C | RAB3C, vesicle exocytosis | 0.397 | 0.021952 |
| FBN2 | Fibrillin 2, elastic fiber assembly | 0.362 | 0.018666 |

| Supplementary Table 2: Pathway / Gene Set | Adjusted P-value |
|---|---|
| Regulation of Cyclin Dependent Protein S/T Kinase Activity (GO) | 0.01 |
| Regulation of Cyclin Dependent Protein Kinase Activity (GO) | 0.01 |
| Vesicle Tethering Complex (GO) | 0.01 |
| Focal Adhesion (KEGG) | 0.02 |
| Cell Projection Membrane (GO) | 0.02 |
| ERK1 and ERK2 Cascade (GO) | 0.02 |
| Arachidonic Acid Metabolism (KEGG) | 0.03 |
| Secretory Granule Membrane (GO) | 0.03 |
| Actin Cytoskeleton (GO) | 0.03 |
| Cortical Actin Cytoskeleton (GO) | 0.03 |
| Regulation of Actin Cytoskeleton (KEGG) | 0.04 |
| PI3K-Akt Signaling Pathway (KEGG) | 0.04 |
| GABAergic Synapse (KEGG) | 0.04 |
| ECM-receptor Interaction (KEGG) | 0.04 |
| Glutamergic Synapse (KEGG) | 0.05 |
| Endocytosis (KEGG) | 0.07 |

**Supplement Table 3: Primary antibodies**

| Antibody | Species | Source, Clone and Cat No | WB | IF |
|---|---|---|---|---|
| Mouse CD36 | Goat | R&D Systems (#AF2519) | 1:100 | 1:50 |
| Human CD36 | Goat | R&D Systems (#AF1955) | 1:100 | 1:50 |
| Caveolin 1 | Rabbit | CST (#3238) | 1:1000 | 1:100 |
| pCaveolin1$^{Y14}$ | Mouse | BD Biosciences (#611338) | 1:1000 | - |
| AKT | Rabbit | CST (pan) (C67E7) (#4691) | 1:1000 | - |
| pAKT$^{S473}$ | Rabbit | CST (D9E) (#4060) | 1:1000 | - |
| Actin | Mouse | SCBT (C4) sc-47778 | 1:1000 | - |
| pERM | Rabbit | CST (#3141) | 1:1000 | - |
| Ceramide | Mouse | Sigma (#C8104) | - | 1:100 |
| Src | Rabbit | CST (36D10) (#2109) | 1:1000 | - |
| Cofilin | Mouse | Sigma (#SAB2702206) | 1:1000 | - |
| pCofilin$^{S3}$ | Rabbit | CST (77G2) (#3313) | 1:1000 | - |
| Rab7 | Rabbit | CST (D95F2) (#9367) | 1:1000 | - |
| CD9 | Rabbit | CST (E8L5J) (#98327) | 1:1000 | - |
| CD81 | Mouse | SCBT(B-11) (#sc-166029) | 1:1000 | - |
| Calnexin | Rabbit | Enzo Life Sciences (#ADI-SPA-860) | 1:1000 | - |
| CD63, hybridoma | Mouse | DSHB | - | 1:2 |

**Supplement Table 4. Primer sequences**

| Primer | Sense | Antisense |
|---|---|---|
| CPT1a | GCAAAAATCAATCGGACTCTGGA | TCAGGGAGTAGCGCATGGT |
| FABP3 | GCGGGAGCTAATTGATGGAA | CTCATAAGTGCGAGTGCAAACTG |
| ACSL3 | AAAACTGGGAGGAAGGTGGATAC | CTTTGGCCCCCAATAAGAATT |
| Glut1 | CAGCAGCCCTAAGGATCTCA | CTACAGTCGGCTCGGCC |
| HK2 | GAGTTTGACCTGGATGTGGTTGC | CCTCCATGTAGCAGGCATTGCT |
| eNOS | ATCTTCAGCCCCAAACGGAG | CTGGAACATCTTCCGCCTGT |
| VEGFR2 | TGGTTGTGTATGTCCCACCC | TACCAGTGGATGTGATGCGG |
| PPARδ | AGAGGAGGAGAAAGAGGAAGTG | ATGAACACCGTAGTGGAAGC |
| IRS1 | AGTCTGTCGTCCAGTAGCACCA | ACTGGAGCCATACTCATCCGAG |
| 36B4 | GTGATGTGCAGCTGATCAAGACT | GATGACCAGCCCAAAGGAGA |
